# Multi-omics data integration reveals novel drug targets in hepatocellular carcinoma

**DOI:** 10.1186/s12864-021-07876-9

**Published:** 2021-08-04

**Authors:** Christos Dimitrakopoulos, Sravanth Kumar Hindupur, Marco Colombi, Dritan Liko, Charlotte K. Y. Ng, Salvatore Piscuoglio, Jonas Behr, Ariane L. Moore, Jochen Singer, Hans-Joachim Ruscheweyh, Matthias S. Matter, Dirk Mossmann, Luigi M. Terracciano, Michael N. Hall, Niko Beerenwinkel

**Affiliations:** 1 Department of Biosystems Science and Engineering, ETH Zürich, 4058 Basel, Switzerland; 2 Swiss Institute of Bioinformatics, Basel, Switzerland; 3 Present address: Roche, PTD Biologics Europe, 4070 Basel, Switzerland; 4 Biozentrum, University of Basel, 4056 Basel, Switzerland; 5 Present address: Novartis Institutes for BioMedical Research, Disease Area Oncology, 4002 Basel, Switzerland; 6 Institute of Pathology, University Hospital Basel, 4031 Basel, Switzerland; 7 Department of BioMedical Research, University of Bern, 3008 Bern, Switzerland; 8 Department of Biomedicine, Visceral Surgery Research Laboratory, Clarunis, Basel, Switzerland; 9 Clarunis Universitäres Bauchzentrum Basel, Basel, Switzerland

**Keywords:** HCC, mTOR signaling, NetICS, Omics

## Abstract

**Background:**

Genetic aberrations in hepatocellular carcinoma (HCC) are well known, but the functional consequences of such aberrations remain poorly understood.

**Results:**

Here, we explored the effect of defined genetic changes on the transcriptome, proteome and phosphoproteome in twelve tumors from an mTOR-driven hepatocellular carcinoma mouse model. Using Network-based Integration of multi-omiCS data (NetICS), we detected 74 ‘mediators’ that relay via molecular interactions the effects of genetic and miRNA expression changes. The detected mediators account for the effects of oncogenic mTOR signaling on the transcriptome, proteome and phosphoproteome. We confirmed the dysregulation of the mediators YAP1, GRB2, SIRT1, HDAC4 and LIS1 in human HCC.

**Conclusions:**

This study suggests that targeting pathways such as YAP1 or GRB2 signaling and pathways regulating global histone acetylation could be beneficial in treating HCC with hyperactive mTOR signaling.

**Supplementary Information:**

The online version contains supplementary material available at 10.1186/s12864-021-07876-9.

## Background

Liver cancer is the second leading cause of cancer-related deaths worldwide, and hepatocellular carcinoma (HCC) accounts for approximately 90% of primary liver cancer cases [1]. Approximately 50% of HCC tumors exhibit loss of the tumor suppressors *Pten*, *Tsc1*, or *Tsc2* leading to aberrant PI3K–AKT–mTOR signaling. However, the effector pathways via which mTOR promotes tumorgenicity are widely unknown. We generated an mTOR-driven HCC mouse model, by liver-specific deletion of the tumor suppressors *Pten* and *Tsc1* [2, 3], to investigate the molecular and cellular mechanisms of mTOR-driven tumorgenicity.

While DNA sequencing has enabled a comprehensive characterization of tumor genomes and stratification of patients, translating such information into treatment strategies has remained a major challenge. A limitation of relying entirely on genomic data to determine a therapeutic strategy is that it ignores functionally-related, non-mutated genes that could also encode potential drug targets. In addition, different mutations across cancer patients (genetic divergence) could result in the same pathways being activated (functional convergence) [4]. Apart from somatic mutations, tumorigenesis can be regulated by the levels of specific miRNAs, mRNAs, proteins and protein phosphorylation. miRNAs are key regulators of the transcriptome and can act as either oncogenes or tumor suppressors. Common mechanisms that can dysregulate miRNA expression in human cancers include amplification, deletion or epigenetic changes [5]. Transcriptomic and proteomic analyses have been performed to stratify HCC patients into clinically-relevant groups [6–8]. However, to further understand the effect of a genetic aberration or dysregulated gene expression (possibly due to aberrant miRNA expression) it is necessary to identify the mediators common to diverse alterations. Distinct genomic aberrations (in different tumors) are expected to converge functionally on the same downstream protein, referred to here as a ‘mediator’. To identify such mediators, it is essential to integrate omics data, i.e., the genome, transcriptome, proteome and phosphoproteome (commonly referred to as multi-omics analysis), from diverse tumors.

Recently, multi-omics analysis has been informative in the characterization of tumors. For example, integration of DNA, RNA and phosphoproteomic data enabled stratification of prostate cancer patients and to identify individualized treatment options [9]. Computational methods that focus on the direct effect of genetic aberrations, i.e., the effect of a gene mutation on the encoded protein, have also been proposed [10]. However, a major drawback of these studies is that they rely solely on genomic analysis. New methods are necessary to integrate different types of omics data to identify dysregulated pathways.

In this study we use NetICS, a computational method to integrate multi-omic data (somatic mutations, miRNA differential expression, transcriptomics, proteomics and phospho proteomics) from an mTOR-driven mouse HCC tumor model [11], to understand the molecular mechanisms of mTOR-driven HCC. NetICS provides a comprehensive framework that reveals how specific genetic aberrations (i.e., deletion of the tumor suppressors *Pten* and *Tsc1*) and tumor-specific changes in miRNA expression can affect downstream mediators. NetICS employs a sample-specific network diffusion process that reveals the convergence of diverse changes in distinct tumors (mutations and differentially expressed miRNAs) on common downstream mediators. The identified mediators include transcription factors, kinases, phosphatases and deacetylases. While, some of the mediators are known oncogenes, others are novel oncogenic mediators. These mediators are potential, novel drug targets.

## Results

**Fig. 1:**
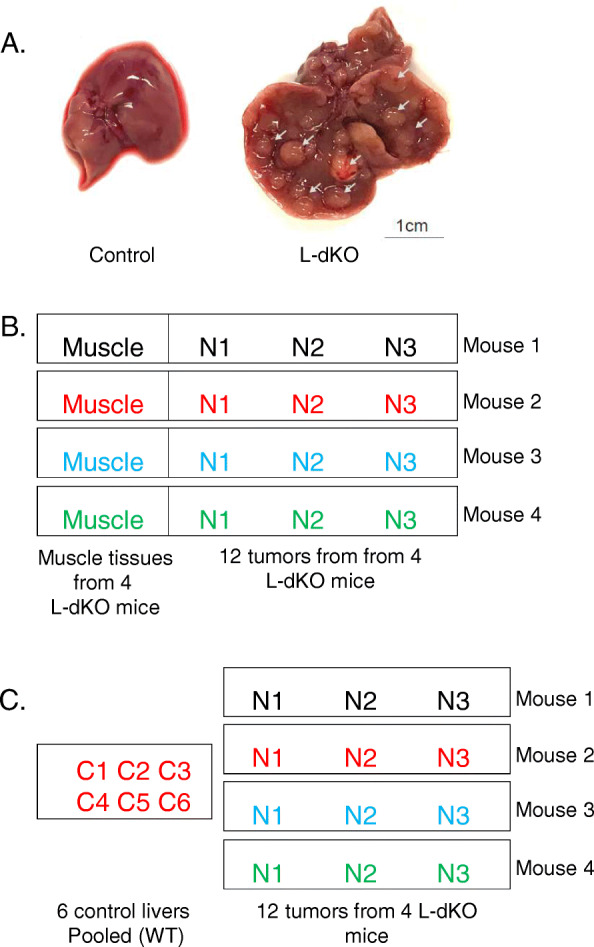
Experimental setting. **A.** Representative images of whole livers from 20-week-old L-dKO (tumors are indicated with arrowheads) and control mice. **B.** Three independent tumor samples were taken from each of four 20-week-old L-dKO mice. For each mouse, one muscle sample was used as the matched healthy tissue. This setting was used for exome sequencing. Each of the three tumor nodules was compared against the matched muscle tissue sample. **C.** Three independent tumor samples were taken from each of four 20-week-old L-dKO mice. Liver tissues from six *cre*-negative age- and sex- matched littermates were used as a control. This setting was used for mRNA sequencing, miRNA sequencing, proteome and phosphoproteome quantification

**Fig. 2:**
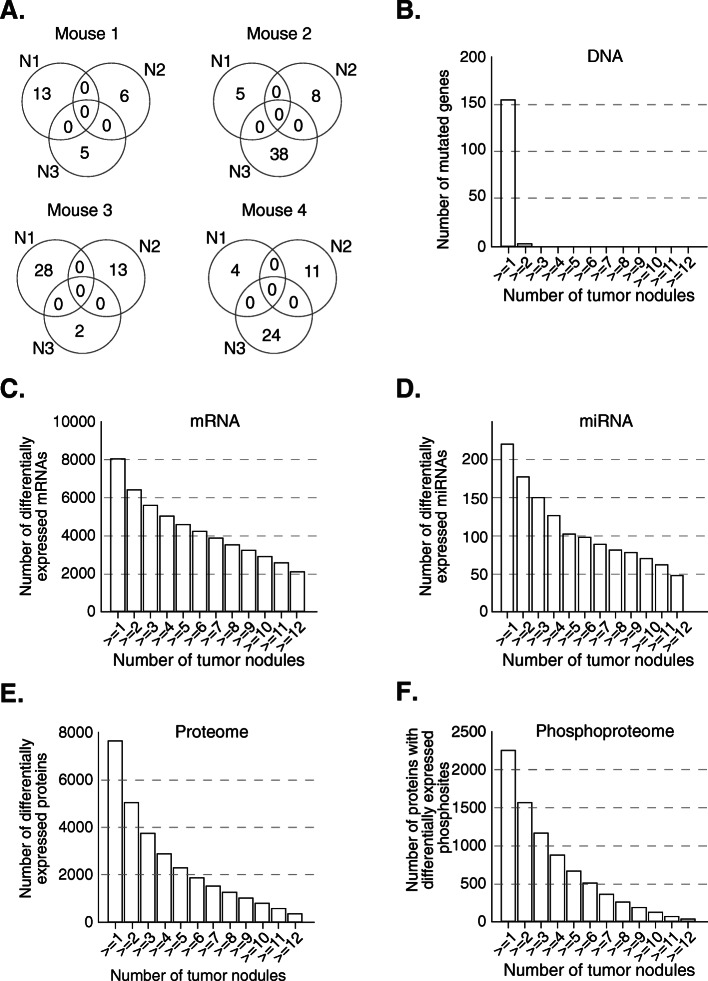
**A.** Shown are Venn diagrams for each of the 4 mice demonstrating the common somatic mutations (point mutations and small insertions/deletions) between the tumor samples of the same mouse. We observe no common mutations between the tumor samples, implying that these are more likely passenger mutations. **B.** Graph showing the number of common mutated genes, differentially expressed mRNAs**, C.**, miRNAs, **D.** proteins, **E.**, and phosphoproteins, **F.**, across the tumor samples as a cumulative histogram. In each of the subfigures, the number of genes or proteins being dysregulated in at least k samples is shown (y-axis), where k ranges from 1 to 12 (x-axis)

**Fig. 3:**
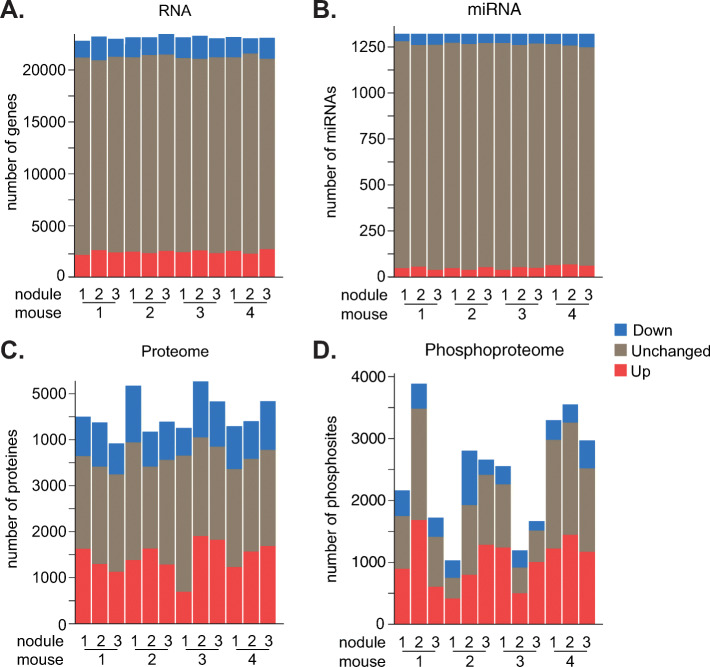
Shown are number of differentially expressed mRNAs, **A.**, and miRNAs, **B.**, proteins, **C.**, and phosphosites, **D.**, in each of the 12 tumor samples. Red color determines up regulation, blue color downregulation and grey color unchanged. mRNAs and miRNAs have been detected from RNA sequencing data and proteins and phosphosites have been detected from mass spectrometry data.

We isolated tumors from an HCC mouse model generated by liver-specific deletion of *Pten* and *Tsc1* **(**Fig. 1A). We hereafter refer to this model as the liver-specific double-knockout (L-dKO) mouse. We isolated twelve distinct liver tumors, three each from four 20 week old L-dKO mice. To detect somatic mutations, we compared exome sequence data from tumors and from matched muscle tissue (Fig. 1B). For all other analyses (RNA, miRNA, proteome and phosphoproteome), we compared the tumor nodules to healthy liver tissue from six control mice (cre-negative, age- and sex-matched littermates) (Fig. 1C). We detected a total of 157 point mutations and small insertions/deletions in the twelve tumors (Table 1). Except for the originally introduced *Pten* and *Tsc1* deletions (Fig. S1), no specific mutation was found in more than a single tumor (Fig. 2A-B). In contrast to somatic mutations, we detected mRNAs, miRNAs, proteins and phosphosites commonly dysregulated across multiple tumor samples (Fig. 2C-F). On average, 4,348 mRNA, 108 miRNAs, 2,389 proteins and 906 phosphosites were dysregulated in the tumor samples (Fig. 3A-D).

**Table 1:** Shown are the results of exome sequencing in the 12 tumor nodules. Relevant information are given such as the position in thechromosome, the reference and alternative alleles, the type of mutation, the amino acid substitution and variant allele frequency

TUMOR_SAMPLE	NORMAL_SAMPLE	CHROM	POS	REF	ALT	GENE	EFFECT	Alteration (cDNA)	Alteration (AA)	Depth in tumor	Variant allele fraction
368N4	368muscle	1	65050991	C	T	Cryge	missense_variant	c.31G>A	p.Gly11Ser	58	8.80%
357N1	357muscle	1	85577158	G	C	G530012D18Rik	missense_variant	c.283G>C	p.Glu95Gln	15	26.70%
368N2	368muscle	1	86426453	C	A	1700019O17Rik	missense_variant	c.83C>A	p.Ala28Asp	42	7.10%
373N4	373muscle	1	92942579	C	A	Capn10	missense_variant	c.786C>A	p.His262Gln	48	6.30%
373N4	373muscle	1	1.06E+08	C	T	Phlpp1	stop_gained	c.4243C>T	p.Arg1415*	110	21.80%
358N3	358muscle	1	1.28E+08	C	A	Lct	missense_variant	c.3054G>T	p.Met1018Ile	43	7.00%
373N4	373muscle	1	1.31E+08	C	A	Pfkfb2	missense_variant	c.619G>T	p.Asp207Tyr	89	4.50%
358N3	358muscle	1	1.66E+08	C	A	Gm4846	missense_variant	c.1305G>T	p.Met435Ile	72	5.60%
358N3	358muscle	1	1.67E+08	G	T	Aldh9a1	missense_variant	c.712G>T	p.Ala238Ser	44	6.80%
368N2	368muscle	1	1.81E+08	C	A	Acbd3	missense_variant	c.337C>A	p.His113Asn	25	12.00%
358N3	358muscle	2	13486755	C	A	Cubn	missense_variant&splice_region_variant	c.480G>T	p.Lys160Asn	47	6.40%
357N1	357muscle	2	25911320	C	A	Kcnt1	synonymous_variant	c.3409C>A	p.Arg1137Arg	35	8.60%
368N2	368muscle	2	58982966	G	T	Ccdc148	missense_variant	c.614C>A	p.Ala205Asp	28	10.70%
358N3	358muscle	2	66565161	C	A	Scn9a	stop_gained	c.538G>T	p.Glu180*	24	12.50%
368N4	368muscle	2	1.04E+08	A	C	D430041D05Rik	missense_variant	c.1942T>G	p.Leu648Val	267	13.10%
373N4	373muscle	2	1.12E+08	A	G	Olfr1308	missense_variant	c.344T>C	p.Leu115Pro	51	11.80%
368N2	368muscle	2	1.2E+08	C	A	Rpap1	missense_variant	c.1903G>T	p.Ala635Ser	34	8.80%
373N4	373muscle	2	1.2E+08	C	A	Pla2g4e	missense_variant	c.665G>T	p.Cys222Phe	38	7.90%
373N3	373muscle	2	1.46E+08	C	A	Cfap61	missense_variant	c.1976C>A	p.Ala659Asp	46	6.50%
368N2	368muscle	2	1.53E+08	C	A	Asxl1	missense_variant	c.3191C>A	p.Pro1064Gln	84	4.80%
368N4	368muscle	2	1.62E+08	A	G	Ptprt	synonymous_variant	c.295T>C	p.Leu99Leu	75	16.00%
368N2	368muscle	2	1.67E+08	C	A	B4galt5	missense_variant	c.766G>T	p.Ala256Ser	16	18.80%
368N2	368muscle	3	86780226	G	T	Lrba	missense_variant	c.8437G>T	p.Ala2813Ser	48	6.30%
358N3	358muscle	3	88360373	G	A	Smg5	synonymous_variant	c.2799G>A	p.Arg933Arg	86	4.70%
357N1	357muscle	3	88367297	G	A	Paqr6	missense_variant	c.685G>A	p.Ala229Thr	22	18.20%
373N3	373muscle	3	1.03E+08	C	A	Csde1	missense_variant	c.845C>A	p.Pro282Gln	47	6.40%
357N5	357muscle	3	1.04E+08	C	A	Rsbn1	missense_variant	c.1871C>A	p.Ala624Asp	27	11.10%
358N3	358muscle	4	24536440	G	T	Mms22l	synonymous_variant	c.2028G>T	p.Ala676Ala	77	5.20%
357N1	357muscle	4	40738329	G	T	Smu1	stop_gained	c.1404C>A	p.Cys468*	35	8.60%
358N3	358muscle	4	41034270	G	T	Aqp7	synonymous_variant	c.888C>A	p.Gly296Gly	17	17.60%
358N1	358muscle	4	56937908	C	A	Tmem245	missense_variant	c.639G>T	p.Leu213Phe	19	15.80%
358N2	358muscle	4	1.01E+08	G	T	Jak1	synonymous_variant	c.1245C>A	p.Leu415Leu	41	7.30%
368N4	368muscle	4	1.26E+08	C	T	Csf3r	synonymous_variant	c.2145C>T	p.Ser715Ser	53	13.20%
358N3	358muscle	4	1.53E+08	G	T	Nphp4	synonymous_variant	c.1500G>T	p.Ser500Ser	46	6.50%
373N4	373muscle	4	1.54E+08	A	G	Cep104	missense_variant	c.1544A>G	p.Lys515Arg	121	4.10%
373N4	373muscle	4	1.55E+08	G	A	Plch2	synonymous_variant	c.3087C>T	p.Ala1029Ala	38	8.10%
373N3	373muscle	4	1.56E+08	A	G	Mib2	synonymous_variant	c.966T>C	p.Ala322Ala	102	5.90%
358N3	358muscle	4	1.56E+08	C	A	Agrn	missense_variant	c.4334G>T	p.Arg1445Leu	46	6.50%
368N2	368muscle	5	34813050	C	A	Htt	missense_variant	c.1541C>A	p.Ser514Tyr	50	6.00%
358N3	358muscle	5	63937830	C	A	Rell1	stop_gained	c.292G>T	p.Glu98*	83	4.80%
357N1	357muscle	5	1.12E+08	C	A	Hps4	synonymous_variant	c.1359C>A	p.Pro453Pro	39	7.70%
373N3	373muscle	5	1.22E+08	C	A	Rad9b	missense_variant	c.727G>T	p.Ala243Ser	40	7.50%
368N2	368muscle	6	29283208	G	T	Fam71f2	missense_variant	c.204G>T	p.Met68Ile	35	8.60%
357N1	357muscle	6	89342587	G	A	Plxna1	missense_variant&splice_region_variant	c.1735C>T	p.Pro579Ser	31	9.70%
358N2	358muscle	6	91486900	C	G	Tmem43	missense_variant	c.1156C>G	p.Pro386Ala	89	5.60%
358N3	358muscle	6	92189633	C	T	Zfyve20	missense_variant	c.2029G>A	p.Ala677Thr	75	20.00%
373N4	373muscle	6	1.29E+08	G	T	BC048546	missense_variant	c.2389C>A	p.Pro797Thr	41	7.30%
358N3	358muscle	7	24710200	C	A	BC049730	missense_variant	c.43C>A	p.Leu15Met	28	10.70%
368N2	368muscle	7	29292075	C	A	Ppp1r14a	missense_variant	c.307C>A	p.Pro103Thr	44	6.80%
368N2	368muscle	7	34204130	T	C	Gpi1	missense_variant	c.1297A>G	p.Thr433Ala	46	6.50%
373N3	373muscle	7	80738221	G	A	Iqgap1	missense_variant	c.2677C>T	p.Arg893Cys	46	6.50%
358N2	358muscle	7	1.18E+08	C	G	Xylt1	missense_variant	c.2763C>G	p.Cys921Trp	38	10.50%
368N4	368muscle	7	1.26E+08	G	A	Gsg1l	synonymous_variant	c.918C>T	p.His306His	121	5.00%
368N2	368muscle	7	1.27E+08	C	A	Zfp768	missense_variant	c.937G>T	p.Gly313Cys	41	7.30%
373N1	373muscle	7	1.4E+08	G	T	Olfr525	missense_variant	c.686G>T	p.Arg229Leu	95	4.20%
368N2	368muscle	7	1.44E+08	C	A	Ppfia1	missense_variant	c.2471G>T	p.Ser824Ile	48	6.30%
373N1	373muscle	8	11517878	C	G	Cars2	missense_variant	c.1216G>C	p.Val406Leu	169	8.30%
368N2	368muscle	8	13955760	C	A	Tdrp	stop_gained	c.160G>T	p.Glu54*	50	6.00%
368N2	368muscle	8	15041975	C	T	BB014433	missense_variant	c.877G>A	p.Val293Met	42	14.30%
358N3	358muscle	8	68358564	G	T	Csgalnact1	missense_variant	c.1453C>A	p.Pro485Thr	50	6.00%
373N4	373muscle	8	72346037	C	A	Eps15l1	missense_variant	c.2509G>T	p.Asp837Tyr	18	16.70%
373N4	373muscle	8	80730168	C	T	Smarca5	synonymous_variant	c.444G>A	p.Glu148Glu	91	5.50%
368N2	368muscle	8	95327967	G	A	Zfp319	missense_variant	c.1607C>T	p.Ala536Val	50	6.00%
358N3	358muscle	8	1.05E+08	G	A	Rrad	missense_variant	c.118C>T	p.Pro40Ser	41	7.30%
358N3	358muscle	8	1.08E+08	G	T	Wwp2	missense_variant	c.1034G>T	p.Arg345Met	43	7.00%
358N3	358muscle	8	1.11E+08	C	A	Fuk	missense_variant	c.970G>T	p.Gly324Cys	40	7.50%
358N3	358muscle	8	1.26E+08	C	A	Ntpcr	synonymous_variant	c.21C>A	p.Leu7Leu	42	7.10%
357N1	357muscle	9	24582820	C	A	Dpy19l2	synonymous_variant	c.2013G>T	p.Val671Val	44	6.80%
358N2	358muscle	9	43311472	G	A	Trim29	synonymous_variant	c.597G>A	p.Leu199Leu	173	4.00%
357N5	357muscle	9	45450529	C	T	Dscaml1	missense_variant	c.586C>T	p.Arg196Cys	50	8.00%
368N8	368muscle	9	55168284	G	A	Ube2q2	missense_variant	c.376G>A	p.Asp126Asn	71	7.00%
368N8	368muscle	9	55168290	C	T	Ube2q2	missense_variant	c.382C>T	p.Pro128Ser	69	7.20%
373N1	373muscle	9	56260482	C	T	Peak1	missense_variant	c.161G>A	p.Arg54Gln	340	2.90%
358N3	358muscle	9	92287625	C	A	Plscr2	missense_variant	c.127C>A	p.Gln43Lys	97	4.10%
357N4	357muscle	9	1.08E+08	G	C	Bsn	missense_variant	c.4976C>G	p.Pro1659Arg	47	21.30%
368N2	368muscle	10	20246611	C	A	Map7	missense_variant	c.422C>A	p.Ala141Asp	37	8.10%
358N3	358muscle	10	20322064	C	G	Bclaf1	missense_variant	c.52C>G	p.Gln18Glu	21	14.30%
368N2	368muscle	10	38966046	C	A	Lama4	missense_variant	c.92C>A	p.Ala31Glu	47	6.40%
358N2	358muscle	10	70534879	G	A	Fam13c	missense_variant	c.848G>A	p.Ser283Asn	40	20.00%
357N5	357muscle	10	80773112	C	A	Dot1l	missense_variant	c.502C>A	p.Gln168Lys	39	7.70%
373N4	373muscle	10	81420600	G	A	Nfic	synonymous_variant	c.229C>T	p.Leu77Leu	150	8.70%
357N1	357muscle	10	1.27E+08	G	T	Mettl1	missense_variant	c.577G>T	p.Asp193Tyr	45	6.70%
373N4	373muscle	11	5707370	G	A	Mrps24	missense_variant	c.148C>T	p.Pro50Ser	97	4.10%
358N3	358muscle	11	60202880	C	A	Srebf1	synonymous_variant	c.2232G>T	p.Ser744Ser	74	5.60%
357N4	357muscle	11	69853226	A	G	Tnk1	missense_variant	c.1306T>C	p.Phe436Leu	114	30.70%
373N3	373muscle	11	78499753	C	A	Vtn	missense_variant	c.237C>A	p.Asp79Glu	48	6.30%
368N4	368muscle	11	87889211	G	A	Olfr462	synonymous_variant	c.684C>T	p.His228His	50	6.00%
357N4	357muscle	11	98250228	A	G	Cdk12	missense_variant	c.4294A>G	p.Lys1432Glu	35	8.60%
368N2	368muscle	11	1.01E+08	A	G	Aoc2	synonymous_variant	c.195A>G	p.Thr65Thr	38	7.90%
368N2	368muscle	11	1.01E+08	A	G	Aoc2	missense_variant	c.269A>G	p.Asn90Ser	35	20.00%
357N4	357muscle	11	1.03E+08	C	G	Fzd2	synonymous_variant	c.1419C>G	p.Leu473Leu	109	28.40%
368N2	368muscle	11	1.08E+08	C	A	Helz	synonymous_variant	c.1356C>A	p.Thr452Thr	32	9.40%
368N2	368muscle	12	4209383	C	A	Adcy3	synonymous_variant	c.2659C>A	p.Arg887Arg	44	9.10%
368N4	368muscle	12	33342134	C	A	Atxn7l1	synonymous_variant	c.771C>A	p.Thr257Thr	85	4.70%
368N4	368muscle	12	70246446	C	A	Trim9	synonymous_variant	c.2310G>T	p.Thr770Thr	67	6.00%
373N1	373muscle	12	72567232	G	A	Pcnxl4	synonymous_variant	c.1950G>A	p.Leu650Leu	47	6.40%
368N2	368muscle	12	82387603	C	A	Sipa1l1	missense_variant	c.2146C>A	p.Gln716Lys	29	10.30%
358N3	358muscle	12	1.02E+08	G	T	Slc24a4	synonymous_variant	c.117G>T	p.Leu39Leu	46	6.50%
368N2	368muscle	12	1.02E+08	C	A	Golga5	missense_variant	c.197C>A	p.Ala66Asp	49	6.10%
358N3	358muscle	13	73672769	G	T	Slc6a18	missense_variant	c.695C>A	p.Ala232Glu	26	11.50%
357N5	357muscle	13	73821238	G	T	Nkd2	missense_variant	c.1108C>A	p.Pro370Thr	46	6.50%
357N1	357muscle	13	93387596	C	A	Homer1	synonymous_variant	c.648C>A	p.Ala216Ala	25	12.00%
373N4	373muscle	14	7945932	G	T	Flnb	missense_variant	c.7336G>T	p.Ala2446Ser	36	8.30%
368N4	368muscle	14	54907149	C	T	Slc22a17	synonymous_variant	c.1128G>A	p.Arg376Arg	99	4.00%
357N1	357muscle	14	55745048	C	A	Dhrs1	missense_variant	c.20G>T	p.Gly7Val	20	15.00%
358N3	358muscle	15	81692128	G	T	Chadl	synonymous_variant	c.2239C>A	p.Arg747Arg	66	6.10%
358N3	358muscle	15	99104471	G	T	Dnajc22	missense_variant	c.996G>T	p.Gln332His	36	8.60%
373N4	373muscle	16	5240002	G	T	Alg1	synonymous_variant	c.837G>T	p.Leu279Leu	63	6.30%
368N4	368muscle	16	14233649	C	A	Myh11	missense_variant	c.1292G>T	p.Arg431Leu	74	5.40%
373N3	373muscle	16	23357761	C	T	St6gal1	missense_variant	c.1103C>T	p.Pro368Leu	155	5.80%
357N1	357muscle	16	45731773	G	T	Abhd10	missense_variant	c.736C>A	p.Gln246Lys	73	5.50%
373N4	373muscle	16	56000642	C	A	Zbtb11	missense_variant	c.2101C>A	p.Gln701Lys	38	7.90%
373N4	373muscle	16	96673771	G	T	Dscam	missense_variant	c.3590C>A	p.Ala1197Glu	47	6.40%
368N2	368muscle	16	97576326	C	A	Tmprss2	missense_variant&splice_region_variant	c.570G>T	p.Lys190Asn	47	6.40%
373N4	373muscle	17	24265204	C	A	Abca17	missense_variant&splice_region_variant	c.4938G>T	p.Lys1646Asn	48	6.30%
358N3	358muscle	17	27101185	C	A	Itpr3	synonymous_variant	c.3009C>A	p.Pro1003Pro	97	5.20%
358N3	358muscle	17	28877021	C	A	Pnpla1	synonymous_variant	c.415C>A	p.Arg139Arg	50	6.00%
373N4	373muscle	17	28982146	T	C	Stk38	synonymous_variant	c.555A>G	p.Thr185Thr	39	25.60%
368N2	368muscle	17	34685203	G	T	Tnxb	missense_variant	c.3686G>T	p.Gly1229Val	48	6.40%
357N4	357muscle	17	80145171	C	A	Galm	missense_variant	c.537C>A	p.Phe179Leu	46	6.50%
368N2	368muscle	18	38259948	G	T	0610009O20Rik	missense_variant	c.1204G>T	p.Ala402Ser	20	15.00%
358N1	358muscle	18	42337039	C	A	Rbm27	missense_variant	c.2900C>A	p.Ser967Tyr	44	6.80%
358N3	358muscle	18	44886378	C	A	Ythdc2	missense_variant	c.4213C>A	p.Pro1405Thr	37	8.10%
368N4	368muscle	19	4733741	G	A	Sptbn2	missense_variant	c.1741G>A	p.Ala581Thr	213	5.20%
373N4	373muscle	19	8896787	A	G	Ints5	synonymous_variant	c.2109A>G	p.Leu703Leu	49	6.10%
373N4	373muscle	19	8896820	C	T	Ints5	synonymous_variant	c.2142C>T	p.Thr714Thr	42	9.50%
368N4	368muscle	19	8978064	G	T	Eef1g	missense_variant	c.1276G>T	p.Val426Leu	24	12.50%
357N1	357muscle	19	34950052	G	T	Kif20b	missense_variant	c.2713G>T	p.Ala905Ser	45	6.70%
373N3	373muscle	19	40072400	G	A	Cyp2c54	missense_variant	c.298C>T	p.Leu100Phe	101	5.00%
373N3	373muscle	19	50225150	G	A	Sorcs1	missense_variant	c.2138C>T	p.Ala713Val	47	6.40%
357N4	357muscle	19	55207920	C	T	Gucy2g	missense_variant	c.2582G>A	p.Arg861His	244	6.10%
358N2	358muscle	19	56851528	C	T	Tdrd1	synonymous_variant	c.2019C>T	p.Asp673Asp	71	9.90%
358N2	358muscle	X	6583974	A	C	Shroom4	missense_variant	c.1187A>C	p.Asn396Thr	26	15.40%
373N3	373muscle	X	20936595	G	A	Elk1	synonymous_variant	c.726C>T	p.Gly242Gly	53	7.50%
358N3	358muscle	X	56501662	G	T	Ddx26b	missense_variant&splice_region_variant	c.1775G>T	p.Gly592Val	43	7.00%
357N1	357muscle	X	1.67E+08	G	T	Tlr7	missense_variant	c.2212C>A	p.Gln738Lys	31	16.10%
357N5	357muscle	17	24267574	GCA	G	Abca17	frameshift_variant	c.4476_4477delTG	p.Ala1493fs	120	3.33%
358N1	358muscle	6	47554188	GTCA	G	Ezh2	disruptive_inframe_deletion	c.558_560delTGA	p.Asp187del	57	5.26%
358N1	358muscle	10	58223101	CA	C	AW822073	frameshift_variant	c.1007delT	p.Leu336fs	74	5.41%
358N1	358muscle	15	78935001	C	CAAG	Nol12	disruptive_inframe_insertion	c.24_26dupGAA	p.Lys9dup	13	30.77%
358N2	358muscle	11	3524692	CGTG	C	Smtn	disruptive_inframe_deletion	c.2115_2117delCAC	p.Thr706del	208	2.88%
358N3	358muscle	2	1.55E+08	GT	G	Itch	splice_donor_variant&intron_variant	c.1430+2delT	.	55	5.45%
358N3	358muscle	4	1.37E+08	GCTT	G	Zbtb40	inframe_deletion	c.2461_2463delAAG	p.Lys821del	134	3.73%
358N3	358muscle	5	1.11E+08	TTGC	T	Ep400	disruptive_inframe_deletion	c.7974_7976delGCA	p.Gln2659del	90	3.33%
358N3	358muscle	5	1.35E+08	ATC	A	Fkbp6	frameshift_variant	c.927_928delGA	p.Glu309fs	84	3.57%
358N3	358muscle	7	1.01E+08	GAC	G	Atg16l2	frameshift_variant	c.972_973delGT	p.Ser325fs	191	3.66%
358N3	358muscle	9	5302474	TC	T	Casp1	frameshift_variant	c.396delC	p.Lys133fs	64	6.25%
358N3	358muscle	10	1.1E+08	TGAA	T	Nav3	disruptive_inframe_deletion	c.5574_5576delTTC	p.Ser1859del	78	3.85%
358N3	358muscle	11	84860577	TG	T	Ggnbp2	frameshift_variant	c.395delC	p.Ala132fs	86	3.49%
358N3	358muscle	16	32793328	AATAG	A	Muc20	frameshift_variant	c.1674_1677delCTAT	p.Tyr559fs	76	3.95%
368N2	368muscle	12	1.04E+08	TAC	T	Serpina3k	frameshift_variant	c.1161_1162delAC	p.Leu387fs	108	3.70%
368N4	368muscle	11	71182505	TGAA	T	Nlrp1b	inframe_deletion	c.508_510delTTC	p.Phe170del	225	1.78%
373N3	373muscle	9	38449182	C	CT	Olfr902	frameshift_variant	c.313dupT	p.Cys105fs	131	3.82%
373N4	373muscle	2	85770217	CTG	C	Olfr1013	frameshift_variant	c.425_426delGT	p.Cys142fs	85	3.53%
373N4	373muscle	11	23745586	TG	T	Rel	frameshift_variant	c.695delC	p.Ser232fs	67	4.48%
373N4	373muscle	12	76609154	GGC	G	Sptb	frameshift_variant	c.4151_4152delGC	p.Arg1384fs	95	4.21%
373N4	373muscle	14	48659272	TCTG	T	Otx2	inframe_deletion	c.325_327delCAG	p.Gln109del	68	4.41%

Shown are the results of exome sequencing in the 12 tumor nodules. Relevant information are given such as the position in the chromosome, the reference and alternative alleles, the type of mutation, the amino acid substitution and variant allele frequency.

To identify the downstream mediators, we used NetICS, a network-based method that integrates multi-omic data to prioritize cancer genes [11]. NetICS provides a framework to simulate how upstream events lead to the dysregulation of downstream genes and proteins. It detects how mediators are dysregulated in each sample, using sample-specific network diffusion. NetICS then systematically integrates the individual ranks to infer a global gene ranking across all tumor samples [11]. However, our NetICS framework failed to predict functional convergence among the 157 detected somatic mutations (except for *Pten* and *Tsc1*) after a random permutation test, suggesting that additional information is required to identify a common downstream mediator. Hence, we integrated the *Pten* and *Tsc1* deletions, the somatic mutations, and the differentially expressed miRNAs in each sample as upstream events. As downstream events, we used the differentially expressed mRNAs, proteins and differentially regulated phosphosites per tumor. After systematic integration, NetICS analysis predicted 74 mediators that are functionally related to differentially expressed miRNA and somatic mutations (upstream) as well as to differentially expressed genes, proteins and phosphosites (downstream) **(**Table 2).

**Table 2:** Genes are ranked based on the mediator score from the final ranked gene list across all tumor nodules. The top 5% ranked genes are shown, excluding the ones with a FDR adjusted *P*-value > 0.05 after the random permutation test. For each gene, the gene name and type are given. For each tumor nodule, +1 denotes upregulation at the RNA, proteome or phosphoproteome levels. Similarly, -1 denotes downregulation. If empty, the gene’s, protein’s or phosphosite’s levels did not change significantly between tumor nodules and control samples

Gene type	RNA(+1 significant upregulation, -1 significant downregulation (compared to normal samples))												PROTEOME(+1 significant upregulation, -1 significant downregulation (compared to normal samples))												PHOSPHOPROTEOME(number of phosphosites upregulated/number of phosphosites downregulated)												
	Mouse1-N1	Mouse1-N2	Mouse1-N3	Mouse2-N1	Mouse2-N2	Mouse2-N3	Mouse3-N1	Mouse3-N2	Mouse3-N3	Mouse4-N1	Mouse4-N2	Mouse4-N3	Mouse1-N1	Mouse1-N2	Mouse1-N3	Mouse2-N1	Mouse2-N2	Mouse2-N3	Mouse3-N1	Mouse3-N2	Mouse3-N3	Mouse4-N1	Mouse4-N2	Mouse4-N3	Mouse1-N1	Mouse1-N2	Mouse1-N3	Mouse2-N1	Mouse2-N2	Mouse2-N3	Mouse3-N1	Mouse3-N2	Mouse3-N3	Mouse4-N1	Mouse4-N2	Mouse4-N3	
phosphatase	-1	-1		-1	-1	-1	-1	-1	-1	-1	-1	-1																									
transcription activator												1	1		1	1		1		1	1	1	1	1										1/0	1/0	1/0	
transcription factor																		-1								1/0								1/0			
transcription factor																																					
kinase										-1							-1			-1				-1								1/0					
kinase						-1																															
transcriptional regulator																																					
kinase		1	1			1	1	1	1	1	1	1								1	1			1													
transcriptional inhibitor		-1	-1				-1		-1																												
kinase		1											1	1	1		1	1		1	1	1	1	1		1/0	1/0					0/1			2/0	1/0	
arginase	-1	-1	-1	-1	-1	-1	-1	-1	-1	-1	-1	-1		-1	-1	-1	-1	-1	-1	-1	-1	-1	-1			0/1			0/2	0/1					0/2		
E3 ubiquitin protein ligase		1		1	1	1	1		1	1		1																									
transcription factor	1	1	1	1	1	1	1	1	1	1	1	1																									
deacetylase		-1		-1		-1	-1																														
transcription factor		-1																						-1													
kinase													-1										1														
growth factor				-1	-1	-1	-1	-1	-1	-1	-1																										
growth factor													-1	-1	-1	-1	-1	-1	-1	-1	-1	-1	-1	-1	0/1	0/1			0/1		0/1			0/1			
transcription factor		-1	-1		-1	-1	-1	-1	-1	-1	-1	-1	1	-1						1	1	1		1		1/0		1/0	1/2	3/0		3/0		3/0	2/0		
kinase															-1		1			1	1								0/1								
transcriptional repressor	-1		-1	-1	-1	-1	-1	-1	-1	-1	-1	-1																									
deacetylase																									1/0				1/0								
kinase																-1				-1																	
methyltransferase	1	1	1	1	1	1	1	1	1	1	1	1								1	1		1	1		1/0											
transcription factor		1		1	1	1	1		1																												
S-transferase	1	1		1	1	1	1	1	1				1	1	1	1	1	1	1	1	1	1	1		1/0	1/0			2/0	1/0							
cyclin	1	1	1	1	1	1	1	1	1	1	1	1	1					1		1																	
chloride intracellular channel	1	1	1	1	1	1	1	1	1	1	1	1	1	1	1		1	1	1	1	1	1	1														
kinase		1		1		1		1	1	1		1	1	1	1	1	1	1	1	1	1	1	1	1		1/0	1/0		1/0						1/0		
kinase	1	1								1	1	1				-1																					
growth factor	1	1	1	1		1	1	1	1	1	1	1	1	1	1		1	1		1	1	1	1	1													
chromatin-binding factor																				-1				1	0/1	0/1											
transcription factor (forkhead family)				-1		-1																				1/0											
kinase			-1					-1	-1										1																		
transcription factor	-1	-1	-1	-1	-1	-1	-1	-1	-1	-1	-1	-1																									
mitochondrial uncoupling proteins	1	1	1	1	1	1	1	1	1	1	1	1																									
growth inhibitory protein		-1		-1	-1	-1	-1	-1	-1	-1	-1	-1												1					0/1					0/2	0/1		
signal transduction proteins																1				1	1			1													
inhibitor metalloproteises	1	1	1	1	1	1	1	1	1	1	1	1																									
aspartic proteases	-1	-1	-1	-1		-1	-1	-1	-1	-1	-1	-1		-1																							
transcription factor																																					
adapter protein	1	1	1	1	1	1	1	1	1	1	1	1	1	1	1	1	1	1	1	1	1	1	1	1					1/0								
Insulin Receptor Substrate	-1	-1	-1	-1	-1	-1	-1	-1	-1	-1	-1	-1																									
transcription factor		1	1	1	1	1	1	1	1	1	1	1																									
Histone-lysine N-methyltransferase						1						1																									
lymphoid-specific helicase	1	1	1	1	1	1	1	1	1	1	1	1						-1																			
anti- and pro-apoptotic regulators					-1																																
kinase																																					
versican proteoglycan	1	1	1	1	1	1	1	1	1	1	1	1																									
transcription factor	-1	-1		-1		-1	-1	-1	-1	-1																										1/0	
spermidine Synthase		1											1	1			1			1	1																
enzyme in polyamine biosynthesis	1	1	1	1	1	1	1	1	1	1	1	1																									
inhibits axol extension																																					
transcription factor		-1	-1																										0/1								
transcription activator	1	1	1	1	1	1	1	1	1	1	1	1																									
secreted mitoattractant	1	1	1	1	1	1	1	1	1	1	1	1																									
Ras protein		1					1										1																				
transcription factor	-1	-1	-1	-1	-1	-1	-1	-1	-1	-1	-1													1													
kinase														1												1/0	1/0			1/0				1/0	1/0	1/0	
transcriptional regulator																																					
chromosomal protein													-1		-1	-1	-1	-1	-1	-1	1		-1		0/1				0/2						1/0		
phosphatase																																					
phosphatase	1	1	1	1	1	1	1	1	1	1	1	1				1														1/0					1/0		
transcription factor		1																																			
acetylhydrolase					1													1					1														
kinase								-1				-1	1				1	1		1	1	1	1		1/0	1/1								1/0	0/1		
vitamin D receptors	-1	-1	-1	-1	-1	-1	-1	-1	-1	-1	-1	-1			-1																						
calcium binding protein															1			1	1		1	1	1	1													
transcription factor																																					
transcriptional modulator	-1	-1	-1				-1	-1	-1																												
Ras protein	1	1	1	1	1	1	1	1	1	1	1	1																									
transcription factor	-1	-1	-1	-1	-1	-1	-1		-1	-1		-1																									
kinase							1	1	1					1	-1	1				1	1	1	1	1		2/0				1/0				1/0	2/0	3/0	
scaffolding protein within caveolar membranes			1	1	1	1		1		1	1	1																									

Genes are ranked based on the mediator score from the final ranked gene list across all tumor nodules. The top 5% ranked genes are shown, excluding the ones with a FDR adjusted *P*-value > 0.05 after the random permutation test. For each gene, the gene name and type are given. For each tumor nodule, +1 denotes upregulation at the RNA, proteome or phosphoproteome levels. Similarly, -1 denotes downregulation. If empty, the gene’s, protein’s or phosphosite’s levels did not change significantly between tumor nodules and control samples.

Pathway enrichment analysis of the mediators indicated a strong enrichment of cellular signaling pathways regulating cell cycle proteins and of TGF☐ signaling, suggesting strong proliferation potential of malignant hepatocytes (Table 3**)**. We also observed an upregulation of epithelial to mesenchymal transition (EMT) factors, suggesting increased metastatic potential. For example, many of the detected mediators are involved in Notch signaling, consistent with the observation that approximately 30% of human HCC displays active Notch signaling [12]. Furthermore, we observed upregulation of IL6 and leptin signaling which have been suggested to play crucial roles in the initiation and development of HCC [13, 14]. Leptin is an important activator of cell proliferation and an inhibitor of cell death. Leptin signaling is also known to have angiogenic effects in multiple cancers including HCC [14]. The 74 mediators, consisting of various different types of regulatory proteins, include 14 kinases, 23 transcription factors, 2 deacetylates and 3 phosphatases. As expected, the mediators include known downstream targets of PTEN and TSC1, such as mTOR, AKT2 and AKT3. The mediators also include known HCC-related proteins, such as YWHAZ [15] and KLF4 [16]. Below, we discuss in detail five of the 74 detected mediators, namely YAP1, GRB2, HDAC4, SIRT1 and LIS1.

**Table 3:** Pathway enrichment results by using Metacore tool

#	Networks	Total	*p*-value	FDR	In Data	Gene names
1	Cell cycle_G1-S Growth factor regulation	195	3.776E-15	2.760E-13	21	AKT3, GRB2, NF-kB, VEGF-A, EGFR, STAT3, c-Myc, Cyclin D, GSK3 alpha/beta, N-Ras, AKT(PKB), NF-kB p50/p50, AKT2, c-Raf-1, GSK3 beta, IRS-1, IGF-1 receptor, AKT1, Cyclin D1, NF-kB1 (p50), SMAD4
2	Cell cycle_G1-S Interleukin regulation	128	3.972E-15	2.760E-13	18	AKT3, GRB2, NF-kB, STAT3, c-Myc, Cyclin D, GSK3 alpha/beta, N-Ras, AKT(PKB), NF-kB p50/p50, AKT2, c-Raf-1, GSK3 beta, Elk-1, IRS-1, AKT1, Cyclin D1, NF-kB1 (p50)
3	Development_Hemopoiesis, Erythropoietin pathway	136	1.868E-13	8.656E-12	17	GRB2, SHIP, NF-kB, STAT3, c-Kit, c-Myc, Cyclin D, N-Ras, AKT(PKB), Bim, NF-kB p50/p50, c-Raf-1, K-RAS, FOXO3A, Elk-1, AKT1, Cyclin D1
4	Signal transduction_NOTCH signaling	235	1.677E-11	5.215E-10	19	GRB2, NF-kB, VEGF-A, NF-kB1 (p105), EGFR, STAT3, c-Myc, AKT(PKB), AKT2, c-Raf-1, Skp2/TrCP/FBXW, GSK3 beta, Cyclin D1, PTEN, mTOR, NF-kB1 (p50), SMAD4, FBXW7, HIF1A
5	Signal Transduction_TGF-beta, GDF and Activin signaling	154	1.876E-11	5.215E-10	16	SIP1 (ZFHX1B), GRB2, NF-kB, EGFR, RUNX2, c-Kit, c-Myc, AKT(PKB), c-Raf-1, IRS-1, IGF-1 receptor, Cyclin D1, mTOR, SMAD4, HIF1A, CREB1
6	Signal transduction_ERBB-family signaling	75	4.616E-11	1.069E-09	12	GRB2, NF-kB, EGFR, STAT3, c-Myc, N-Ras, AKT(PKB), c-Raf-1, K-RAS, Elk-1, IRS-1, PTEN
7	Development_EMT_Regulation of epithelial-to-mesenchymal transition	224	6.898E-11	1.254E-09	18	SIP1 (ZFHX1B), GRB2, EGFR, TCF8, STAT3, EGR1, ROCK1, AKT(PKB), c-Raf-1, GSK3 beta, Elk-1, TNF-alpha, CTGF, PTEN, mTOR, SMAD4, HIF1A, CREB1
8	Inflammation_IL-6 signaling	119	7.216E-11	1.254E-09	14	AKT3, GRB2, NF-kB, STAT3, c-Myc, AKT(PKB), NF-kB p50/p50, AKT2, c-Raf-1, 14-3-3 zeta/delta, Elk-1, AKT1, NF-kB1 (p50), 14-3-3
9	Signal transduction_Leptin signaling	107	2.434E-10	3.760E-09	13	GRB2, NF-kB, VEGF-A, STAT3, EGR1, GSK3 alpha/beta, AKT(PKB), AKT2, c-Raf-1, IRS-1, AMPK alpha subunit, HIF1A, CREB1
10	Cardiac development_Role of NADPH oxidase and ROS	134	3.626E-10	4.742E-09	14	GRB2, HDAC5, MEF2C, NF-kB, GSK3 alpha/beta, AKT(PKB), TBX3, c-Raf-1, GSK3 beta, SMAD5, Hamartin, PTEN, SMAD4, HIF1A
11	Reproduction_FSH-beta signaling pathway	160	3.752E-10	4.742E-09	15	NF-kB, VEGF-A, EGFR, EGR1, c-Myc, Cyclin D, AKT(PKB), c-Raf-1, IRS-1, IGF-1 receptor, CTGF, mTOR, SMAD4, HIF1A, CREB1
12	Signal transduction_Androgen receptor signaling cross-talk	72	5.284E-10	6.120E-09	11	GRB2, NF-kB, EGFR, STAT3, AKT(PKB), c-Raf-1, FOXO3A, GSK3 beta, IGF-1 receptor, mTOR, CREB1
13	Inflammation_Amphoterin signaling	118	8.432E-10	9.015E-09	13	ROCK, AKT3, NF-kB, NF-kB1 (p105), ROCK1, AKT(PKB), NF-kB p50/p50, AKT2, c-Raf-1, Elk-1, AKT1, TNF-alpha, NF-kB1 (p50)
14	Signal transduction_ESR1-membrane pathway	91	6.908E-09	6.859E-08	11	GRB2, EGFR, GSK3 alpha/beta, AKT(PKB), c-Raf-1, GSK3 beta, Elk-1, IRS-1, IGF-1 receptor, Cyclin D1, CREB1
15	Inflammation_TREM1 signaling	145	1.087E-08	1.007E-07	13	AKT3, GRB2, MEF2C, NF-kB, EGR1, AKT(PKB), AKT2, c-Raf-1, 14-3-3 zeta/delta, Elk-1, AKT1, TNF-alpha, 14-3-3
16	Translation_Regulation of initiation	127	2.325E-08	1.968E-07	12	AKT3, GRB2, EGFR, GSK3 alpha/beta, AKT(PKB), AKT2, c-Raf-1, GSK3 beta, IRS-1, Hamartin, AKT1, mTOR
17	Signal transduction_ESR1-nuclear pathway	216	2.407E-08	1.968E-07	15	GRB2, VEGF-A, NF-kB1 (p105), EGFR, c-Myc, AKT(PKB), AKT2, c-Raf-1, GSK3 beta, IRS-1, HDAC4, IGF-1 receptor, Cyclin D1, NF-kB1 (p50), SMAD4
18	Development_Hedgehog signaling	253	2.929E-08	2.262E-07	16	GRB2, VEGF-A, TCF8, EGR1, c-Myc, Sirtuin1, ROCK1, c-Raf-1, Skp2/TrCP/FBXW, GSK3 beta, SMAD5, AKT1, Cyclin D1, SMAD4, FBXW7, CREB1
19	Development_Regulation of angiogenesis	222	3.480E-08	2.546E-07	15	GRB2, NF-kB, VEGF-A, EGFR, TCF8, STAT3, c-Myc, AKT(PKB), c-Raf-1, CTGF, SMAD4, HIF1A, IGFBP7/8, CREB1, VEGFR-1
20	Immune response_BCR pathway	137	5.467E-08	3.800E-07	12	GRB2, SHIP, NF-kB, EGR1, GSK3 alpha/beta, AKT(PKB), NF-kB p50/p50, c-Raf-1, Elk-1, PTEN, mTOR, NF-kB1 (p50)
21	Signal transduction_Nitric oxide signaling	88	6.433E-08	4.258E-07	10	NF-kB, VEGF-A, AKT(PKB), NF-kB p50/p50, c-Raf-1, Elk-1, IRS-1, CaMK II alpha, TNF-alpha, CREB1
22	Inflammation_IL-13 signaling pathway	91	8.902E-08	5.624E-07	10	GRB2, STAT3, ARG1, c-Myc, AKT(PKB), c-Raf-1, Elk-1, IRS-1, NF-kB1 (p50), CREB1
23	Immune response_TCR signaling	174	9.726E-08	5.669E-07	13	ROCK, GRB2, NF-kB, NF-kB1 (p105), ROCK1, AKT(PKB), Bim, NF-kB p50/p50, c-Raf-1, Elk-1, AKT1, TNF-alpha, NF-kB1 (p50)
24	Cell cycle_G2-M	206	9.789E-08	5.669E-07	14	AKT3, GRB2, EGFR, c-Myc, AKT(PKB), AKT2, DNMT1, c-Raf-1, Skp2/TrCP/FBXW, 14-3-3 zeta/delta, HDAC4, IGF-1 receptor, AKT1, 14-3-3
25	Cardiac development_FGF_ErbB signaling	124	1.804E-07	1.003E-06	11	GRB2, MEF2C, EGFR, FOG2, Neurofibromin, AKT(PKB), TBX3, c-Raf-1, GSK3 beta, Versican, Hamartin
26	Inflammation_IL-10 anti-inflammatory response	87	6.923E-07	3.701E-06	9	NF-kB, STAT3, c-Myc, Cyclin D, AKT(PKB), NF-kB p50/p50, Cyclin D1, TNF-alpha, NF-kB1 (p50)
27	Inflammation_IL-4 signaling	115	8.212E-07	4.228E-06	10	GRB2, SHIP, NF-kB, AKT(PKB), Bim, c-Raf-1, GSK3 beta, Elk-1, IRS-1, PTEN
28	Signal Transduction_BMP and GDF signaling	91	1.018E-06	5.054E-06	9	RUNX2, c-Myc, AKT(PKB), YY1, AKT2, SMAD5, AKT1, SMAD4, CREB1
29	Apoptosis_Anti-Apoptosis mediated by external signals via PI3K/AKT	233	2.791E-06	1.338E-05	13	GRB2, NF-kB, VEGF-A, EGFR, AKT(PKB), Bim, NF-kB p50/p50, FOXO3A, IRS-1, IGF-1 receptor, TNF-alpha, NF-kB1 (p50), VEGFR-1
30	Inflammation_IL-2 signaling	104	3.152E-06	1.461E-05	9	GRB2, NF-kB, STAT3, AKT(PKB), NF-kB p50/p50, c-Raf-1, Elk-1, PTEN, NF-kB1 (p50)
31	Proliferation_Positive regulation cell proliferation	221	9.263E-06	4.154E-05	12	GRB2, VEGF-A, EGFR, STAT3, c-Kit, c-Myc, AKT(PKB), c-Raf-1, GSK3 beta, IGF-1 receptor, Cyclin D1, VEGFR-1
32	Cardiac development_Wnt_beta-catenin, Notch, VEGF, IP3 and integrin signaling	151	9.785E-06	4.250E-05	10	MEF2C, VEGF-A, GSK3 alpha/beta, TBX3, c-Raf-1, GSK3 beta, Versican, MEF2, PTEN, VEGFR-1
33	Development_Blood vessel morphogenesis	228	1.272E-05	5.358E-05	12	GRB2, NF-kB, VEGF-A, EGFR, STAT3, c-Myc, AKT(PKB), c-Raf-1, CTGF, HIF1A, IGFBP7/8, VEGFR-1
34	Reproduction_Feeding and Neurohormone signaling	210	3.159E-05	1.292E-04	11	NF-kB, STAT3, c-Kit, c-Myc, AKT(PKB), c-Raf-1, Elk-1, TNF-alpha, mTOR, HIF1A, CREB1
35	Proliferation_Negative regulation of cell proliferation	184	5.446E-05	2.163E-04	10	GRB2, KLF4, EGR1, Neurofibromin, c-Myc, Mxi1, c-Raf-1, Elk-1, IGF-1 receptor, Cyclin D1
36	Immune response_IL-5 signalling	38	7.474E-05	2.886E-04	5	GRB2, STAT3, c-Myc, AKT(PKB), c-Raf-1
37	Muscle contraction_Nitric oxide signaling in the cardiovascular system	124	9.686E-05	3.639E-04	8	MEF2C, VEGF-A, Sirtuin1, AKT(PKB), AMPK alpha 1 subunit, Elk-1, HIF1A, CREB1
38	Apoptosis_Anti-Apoptosis mediated by external signals by Estrogen	95	1.178E-04	4.309E-04	7	GRB2, c-Myc, AKT(PKB), c-Raf-1, Elk-1, NF-kB1 (p50), CREB1
39	Development_ERK5 in cell proliferation and neuronal survival	24	1.597E-04	5.691E-04	4	MEF2C, c-Myc, c-Raf-1, CREB1
40	Proliferation_Lymphocyte proliferation	210	1.643E-04	5.711E-04	10	AKT3, GRB2, NF-kB, STAT3, AKT(PKB), AKT2, c-Raf-1, AKT1, TNF-alpha, mTOR
41	Cell adhesion_Integrin-mediated cell-matrix adhesion	214	1.918E-04	6.280E-04	10	ROCK, GRB2, Caveolin-2, c-Myc, ROCK1, AKT(PKB), c-Raf-1, GSK3 beta, Hamartin, Cyclin D1
42	Reproduction_Progesterone signaling	214	1.918E-04	6.280E-04	10	GRB2, VEGF-A, EGFR, STAT3, c-Myc, c-Raf-1, GSK3 beta, IGF-1 receptor, AKT1, CREB1
43	Inflammation_IgE signaling	137	1.943E-04	6.280E-04	8	GRB2, NF-kB, AKT(PKB), NF-kB p50/p50, c-Raf-1, Elk-1, TNF-alpha, NF-kB1 (p50)
44	Inflammation_Neutrophil activation	215	1.992E-04	6.294E-04	10	ROCK, GRB2, NF-kB, STAT3, ROCK1, AKT(PKB), NF-kB p50/p50, c-Raf-1, TNF-alpha, NF-kB1 (p50)
45	Inflammation_MIF signaling	140	2.256E-04	6.967E-04	8	GRB2, NF-kB, Cyclin D, NF-kB p50/p50, Cyclin D1, TNF-alpha, NF-kB1 (p50), CREB1
46	Apoptosis_Anti-Apoptosis mediated by external signals via MAPK and JAK/STAT	179	2.375E-04	7.176E-04	9	GRB2, STAT3, EGR1, c-Myc, Bim, c-Raf-1, Elk-1, TNF-alpha, CREB1
47	Inflammation_Protein C signaling	108	2.628E-04	7.606E-04	7	ROCK, NF-kB, ROCK1, AKT(PKB), NF-kB p50/p50, TNF-alpha, NF-kB1 (p50)
48	Signal transduction_ESR2 pathway	77	2.727E-04	7.606E-04	6	GRB2, VEGF-A, EGFR, c-Raf-1, AKT1, TNF-alpha
49	Apoptosis_Apoptosis stimulation by external signals	144	2.736E-04	7.606E-04	8	GRB2, SHIP, NF-kB, AKT(PKB), Bim, c-Raf-1, TNF-alpha, SMAD4
50	Development_Skeletal muscle development	144	2.736E-04	7.606E-04	8	HDAC5, MEF2C, VEGF-A, Sirtuin, Histone deacetylase class II, Sirtuin1, HDAC4, MEF2
Info						
General						
**Date**	21/07/2018					
**Server**	portal.genego.com					
**Version**	6.35.69300					
Workflow Settings						
**Type**	Enrichment analysis					
**Signals**	both					
Active Data						
**Name**	**Type**	**Size**				
74_NetICS_mouse_data_hub_19_Jul_2018_genelist	General	74				

Shown are pathway enrichment results generated by using Metacore version 6.35.69300 at 21.07.2018. The final list of predicted genes from NetICS were used (Table 2). Pathways are ranked based on the adjusted FDR *P*-value (column E) and the number and names of the pathway genes that are present in the final list of 74 predicted genes is given (column F-G).

**Fig. 4:**
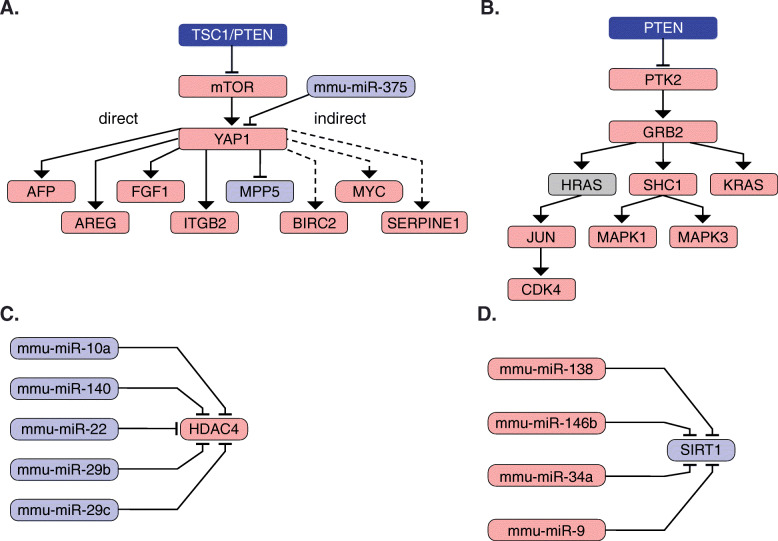
**A.** Aberrant upstream and downstream interactors of YAP1. Upstream interactors include one significantly downregulated miRNAs (miR-375) and interaction with TSC1 and PTEN. Downstream, YAP1 interacts directly or indirectly with several genes the RNA expression of which has been found significantly upregulated. **B.** Aberrant upstream and downstream interactors of GRB2 are shown. Upstream interactors include an indirect interaction with PTEN (through PTK2). Downstream, GRB2 interacts with several genes like SHC1, K-RAS, JUN and CDK4. **C.** miRNAs upstream of HDAC4 significantly downregulated in HCC tumors compared to the control tissue. **D.** miRNAs upstream of SIRT1 significantly upregulated in HCC tumors compared to the control tissue. The expression counts have been normalized with respect to library sizes and have been transformed for variance stabilization. Shown are normalized expression counts of the miRNAs upstream of *Sirt1*. The expression counts have been normalized in the same way as in C. Blue color indicates downregulated or genomic deletion, grey color indicates not regulated and red color indicates upregulation

**Figure 5:**
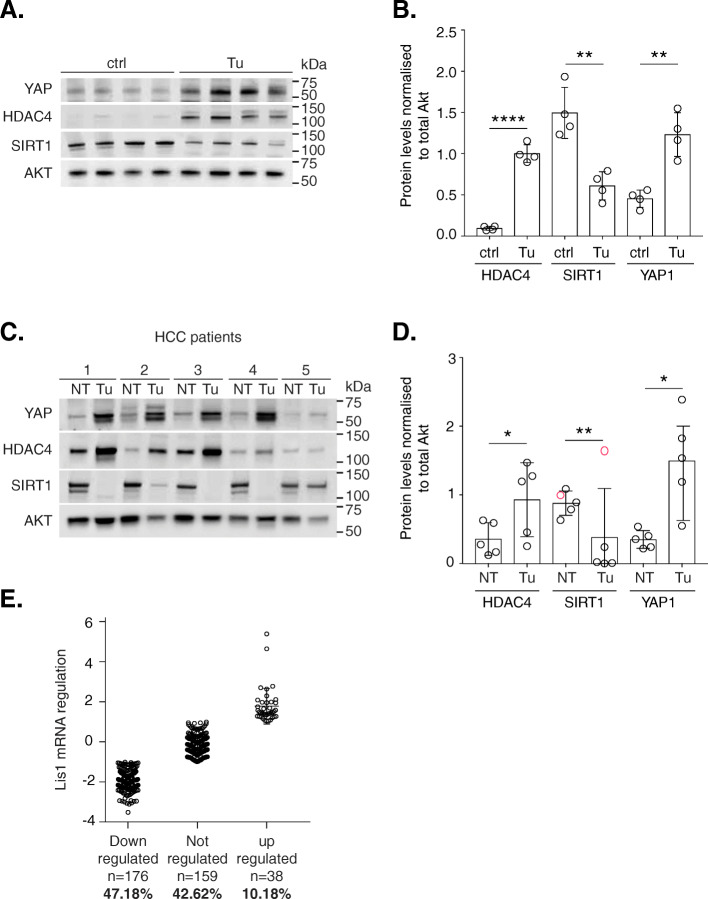
**A.** Immunoblot analysis indicates increased *Hdac4* and *Yap1* and reduced *Sirt1* protein levels in L-dKO tumors (*n* = 4) compared to age-matched littermate control (liver samples from control mice (*n* = 4)). **B.** Quantification of immunoblot (from Fig. 5A) indicates increased *Hdac4* (****P = 0.000004) and increased *Yap1* (**P = 0.0016) and reduced *Sirt1* (**P = 0.0025) expression in tumors compared to age-matched control littermates (band intensities in each lane are normalized to intensity of corresponding total *Akt* protein levels). *P* values are from a two-sided unpaired t-test. Data is mean ± s.d. **C.** Immunoblot analysis indicates increased *Hdac4* and *Yap1* and reduced *Sirt1* protein levels in liver tissue from patients with HCC compared to adjacent non- tumor liver tissue in a total of *n* = 5 HCC patients. **D.** Quantification of immunoblot (from Fig. 5C) indicates increased Hdac4 (*P = 0.040032) and increased Yap1 (*P = 0.013164) in 5 out of 5 patients and reduced Sirt1 (**P = 0.007155) in 4 out of 5 patients. 1 out of 5 patients did not show a reduction in Sirt1 protein levels (circles in red) and has been excluded in the significance analysis. (band intensities in each lane are normalized to intensity of corresponding total *Akt* protein levels). *P* values are from a two-sided paired t-test. Data are mean ± standard deviation. **E.** Lis1 mRNA is downregulated in liver cancer. Graphical representation of Lis1 mRNA regulation in *n*=373 liver cancer patients downloaded from TCGA (provisional) (accessed on 10.10.2018). Each circle represents a patient. 2 fold regulation of mRNA expression compared to control was used to define up regulation (log2 fold change ≥ 1) or down regulation (log2 fold change ≤ 1). Lis1 mRNA expression is downregulated in 47%, unchanged in 43% and upregulated in 10% of the liver cancer patients. The full length images are shown in supplementary figure 5 (Figure S5)

YAP1 is a transcription factor that activates genes involved in cell proliferation and suppresses apoptotic genes [17]. Directly upstream of YAP1 is the microRNA miR-375, the expression of which was significantly downregulated in L-dKO tumors compared to control liver tissues (Fig. 4A and S2A). miR-375 has been shown to inhibit the expression of YAP1 [18]. Our data suggests that the reduced expression of miR-375 could in turn increase YAP1 protein levels that promote tumorigenesis. We also investigated multiple proteins whose expression is regulated by YAP1 (directly or indirectly). As expected, the transcript levels of YAP1 targets were significantly high in HCC tumors, including mRNAs of the direct YAP1 targets *Ctgf*, *Birc5*, *Cyce1*, *Cyr61*, *Ki63* (Fig. S2B). Increased YAP1 levels upon immunoblot analysis in murine (4 out of 4 tumors) and human HCC (5 out of 5 patients) confirmed the dysregulation of YAP1 signaling in HCC (Fig. 5A-D).

The signaling adaptor protein GRB2 was found to be upregulated at both the mRNA and protein level in L-dKO tumors. According to the network (Fig. 4B), GRB2 upregulation can be attributed to downregulation of PTEN. Consistent with the previous observation that PTEN inhibits PTK2 [19], loss of PTEN in the L-dKO tumors correlates with upregulation of PTK2-GRB signaling. GRB2 signaling activates several proteins including SHC1, K-RAS and H-RAS (Fig. 4B). H-RAS is a small GTPase that positively controls phosphorylation of the transcription factor JUN. Mass spectrometry analysis showed that phosphorylation of Ser63 and Ser73 (indicating active JUN) in JUN was significantly increased in L-dKO tumors. JUN in turn regulates transcription of the gene *CDK4.* CDK4 transcript levels and protein levels were upregulated in L-dKO tumors. CDK4 is a known oncoprotein that can be targeted by inhibitors [20]. SHC1 activates MAPK1 and MAPK3, two known protein-serine/threonine kinases that participate in the RAS-RAF-MEK-MAPK signal transduction cascade and are known to be involved in tumorigenesis [21].

NetICS also detected two deacetylases, namely HDAC4 (class II histone deacetylase) and SIRT1 (class III histone deacetylase) as mediators. HDAC4 is known to mediate tumorigenesis through chromatin structure remodeling and controlling protein access to DNA in colon cancer [22], glioblastoma [23], ovarian cancer [24], gastric cancer [18], and esophageal carcinoma [25]. Immunoblot analysis confirmed that HDAC4 protein levels were significantly increased in murine and human HCC tumor tissues (Fig. 5A-D). These observations also suggest that mechanisms similar to mouse L-dKO tumors (i.e., miRNAs) could be regulating HDAC4 protein levels in human HCC. NetICS analysis suggested that five significantly downregulated miRNAs (miR-10a, miR-140, miR-22, miR-29b and miR-29c) could lead to increased HDAC4 levels in tumors (Fig. 4C and S3). SIRT1 is another histone deacetylase detected as a mediator. Immunoblot analysis revealed that SIRT1 protein levels were significantly reduced in murine and human (four of five patients) HCC (Fig. 5A-D). The full length images are shown in supplementary figure 5 (Figure S5). However, unlike HDAC4, SIRT1 mRNA levels were reduced in four out of twelve L-dKO tumors. Upstream of SIRT1, NetICS detected four miRNAs that were significantly upregulated, namely miR-138, miR-146b, miR-34a and miR-9, that could contribute to reduced SIRT1 levels (Fig. 4D and S4). The role of SIRT1 in tumorigenesis is debated due to conflicting reports on SIRT1 as a tumor promoter or suppressor. SIRT1 deacetylates and downregulates two well-known tumor suppressors, TP53 and E2F1, suggesting an oncogenic role [26]. Conversely, SIRT1 also deacetylates and represses the oncogenic transcription factor β-catenin, suggesting a role as a tumor suppressor [27]. Based on our analysis, we suggest that SIRT1 has a tumor suppressing role in mTOR-driven HCC tumors.

NetICS also detected proteins largely unexplored in cancer biology as mediators of tumorigenesis. For example, LIS1 (lissencephaly-1) is a conserved regulator of dynein. It binds to dynein’s motor domain and induces a tight microtubule-dynein interaction [28]. A potential role of LIS1 in tumor progression is now being explored [29, 30]. We examined TCGA transcriptome data for LIS1 expression. We found that 47.1% of HCC patients have reduced LIS1 expression, suggesting that LIS1 has a tumor suppressing role in mTOR-driven tumors (Fig. 5E).

## Discussion

We have utilized NetICS, a multi-omics data integration method that predicts mediators, and an mTOR-driven HCC mouse model to detect novel drug targets in HCC. NetICS detected 74 mediators that were ranked in the top 5% among network proteins. These mediators were found to be significant after a random permutation test of the aberrant and differentially expressed genes and proteins. We described five of the mediators in detail, namely YAP1, GRB2, HDAC4, SIRT1, and LIS1, and suggest upstream causes of their dysregulation as well as their downstream effects.

Importantly, NetICS is able to predict ‘silent’ genes as mediators, i.e., genes not affected by mutation or differentially expressed **(**Table 2**)**. This could be because NetICS scans the neighborhood of the potential mediator and detects aberrant expression and mutation patterns even if the gene itself is neither mutated nor aberrantly expressed. To demonstrate the power of NetICS approach, we tested the ability of multi-omic NetICS to detect mediators that would not be predicted in single-omic approaches, i.e. RNA, proteome or phosphoproteome data alone. Of the 74 top 5%-ranked mediators detected in the multi-omics NetICS approach, we detected 12 relying exclusively on the transcriptome, and none relying only on the proteome or phosphoproteome **(**Table 4**)**. Thus, NetICS has power in predicting silent genes that would not be detected by a single-omics approach.

**Table 4:** Tumor samples are compared against control samples at the RNA (column B), proteome (column C) and phosphoproteome (column D) levels for the 74 predicted mediators. The gene is indicated as “dysregulated”, if it is ranked at the top 5% of all genes based on *P*-value

Gene	RNA all vs all, top 5%	PROT all vs all, top 5%	PHOSPHOPROTEOME all vs all, top 5%
PTEN			
STAT3			
NFKB1			
TNF			
AKT1			
IGF1R			
HIF1A			
CAMK2A			
ZEB2			
GSK3B	dysregulated		
ARG1			
FBXW7			
RUNX1			
SIRT1			
ZFPM2			
AKT2			
VEGFA	dysregulated		
EGFR			
YAP1			
ROCK1			
MXI1			
HDAC4			
AKT3	dysregulated		
DNMT1			
MYC			
GSTM1			
CCND1	dysregulated		
CLIC5			
PRKAA1			
FLT1			
GRB2			
ATXN1			
FOXO3			
KIT	dysregulated		
TBX3			
UCP2			
TSC1			
SMAD4			
TIMP3			
BACE1			
RUNX2	dysregulated		
YWHAZ	dysregulated		
IRS1			
KLF4			
EZH2	dysregulated		
HELLS			
BCL2L11			
AKT	dysregulated		
VCAN			
NF1			
SRM			
AMD1			
SEMA4B			
ZEB1			
MEF2C	dysregulated		
CTGF			
KRAS			
CREB1			
RIOK3			
EGR1			
MECP2			
ENPP6	dysregulated		
INPP5D			
ELK1			
PAFAH1B1			
MTOR	dysregulated		
NR1I3			
CAB39			
MEOX2			
SMAD5			
NRAS			
YY1			
RAF1			
CAV2			

Tumor samples are compared against control samples at the RNA (column B), proteome (column C) and phosphoproteome (column D) levels for the 74 predicted mediators. The gene is indicated as “dysregulated”, if it is ranked at the top 5% of all genes based on *P*-value.

Pathway enrichment on the detected mediator genes suggested multiple tumor-related pathways that could be potentially targeted to curb tumor growth. We focused on the mechanistic insights and pathways of 5 of these mediators that we picked manually. NetICS suggests that overexpression of HDAC4 - which is frequently dysregulated in human malignancies - drives tumor growth in HCC. Inhibitors of HDAC4, such as LMK-235 [31], could be potentially useful in HCC with HDAC4 overexpression. Similarly, HCC with YAP1 overexpression could benefit from using inhibitors for YAP1 [32].

## Conclusions

To conclude, application of NetICS to multi-omics data from an mTOR-driven HCC mouse model detected new potential drug targets. This approach could be used to identify drug targets in other tumor types.

## Methods

### Animal experiments

Liver-specific Tsc1 and Pten double knockout mice were generated as described in [2] and [3] at Biozentrum, University of Basel. In short, tumors from 20 week-old L-dKO mice and whole liver from control mice were snap-frozen and pulverized. This powder was used for subsequent exome sequencing, total RNA sequencing (including miRNA and mRNA), proteomics and phosphoproteomics. For exome sequencing, muscle tissue from the quadriceps of 4 L-dKO mice was used as a control. The mice were on mixed genetic background (C57BL/6J, 129/SvJae, BALB/cJ). Age and sex matched littermate mice without the Cre gene were used as controls. Only male mice were used in all experiments. Mice were fasted overnight before euthanasia by CO2 inhalation. The total number of mice used were 6 control mice and 4 L-dKO mice.

### Exome sequencing

DNA extracted from three tumor nodules and a muscle tissue sample each from four mice were subjected to whole-exome capture using the SureSelect Mouse All Exon (Agilent) capture system and to massively parallel sequencing on an Illumina HiSeq 2000 at the Genomics Facility Basel, ETH Zurich, Switzerland. A median of 141 and 97 million 101-bp paired-end reads were generated from DNA extracted from tumor nodules and the muscle, respectively, equivalent to median depths of 78x (tumor nodules, range 34x-124x) and 57x (germline, range 35x-129x; Table 5). Exome sequencing data have been deposited in the Sequence Read Archive under the accession SRP156216.

**Table 5:** Statistics of whole-exome sequencing

SAMPLE	Total number of reads	Mean Target Coverage	% target bases covered at least 10X	% target bases covered at least 20X	% target bases covered at least 50X	% target bases covered at least 100X
357muscle	55,062,491	35.5	88.9%	66.7%	19.6%	3.3%
368muscle	68,408,757	43.6	92.1%	75.1%	28.2%	5.8%
373muscle	125,208,792	69.7	96.1%	88.5%	54.2%	18.3%
358muscle	249,603,786	128.7	98.0%	95.4%	80.1%	49.2%
358N1	52,236,649	33.8	88.1%	64.8%	18.1%	3.0%
357N5	59,870,967	37.5	90.0%	69.5%	22.3%	4.1%
368N8	69,492,339	43.2	91.9%	75.0%	28.6%	6.1%
373N4	92,890,008	55.4	94.0%	82.4%	42.4%	12.2%
368N2	126,289,004	63.6	95.8%	87.2%	50.1%	15.5%
357N1	140,082,301	77.6	96.2%	89.4%	59.0%	23.8%
358N3	144,107,456	78.4	96.5%	90.2%	61.2%	25.2%
373N3	141,719,445	84.8	96.7%	91.0%	64.2%	28.4%
357N4	172,106,294	91.2	97.2%	92.4%	67.3%	31.1%
368N4	207,370,862	110.2	97.9%	94.8%	76.5%	42.3%
358N2	209,306,186	113.7	97.8%	94.5%	76.3%	43.0%
373N1	235,568,319	123.9	97.9%	95.0%	79.0%	47.9%

Statistics about whole-exome sequencing are given. These include the total number of reads, the mean target coverage and the percent of target bases covered at least at 10X, 20X, 50X and 100X for each tumor and muscle tissue sample.

Whole-exome sequencing data pre-processing was performed as described in Nuciforo et al, 2018 against the reference mouse genome GRCm38. In brief, paired-end reads in FASTQ format were aligned to the reference mouse genome GRCm38 using Burrows-Wheeler Aligner (v0.7.12) [33]. Local realignment was performed using the Genome Analysis Toolkit (GATK, v3.6) [34]. PCR duplicates were removed using Picard (v2.4.1, http://broadinstitute.github.io/picard/). Base quality adjustment was performed using GATK (v3.6) [34].

Somatic single-nucleotide variants (SNVs) were identified using MuTect (v1.1.4) [35] and somatic small insertions and deletions (indels) were identified using Strelka (v1.0.15) [36]. To remove false mutation calls resulting from sequencing and/or alignment artifacts, a panel of normal was created from the four normal samples in this cohort using the artifact detection mode of MuTect2 (packaged in GATK, v3.6). Variants present in at least two of the four samples in the panel of normal were disregarded. Variants outside the target regions, covered by <10 reads in the tumor or <5 reads in the germline were disregarded. Variants supported by <3 reads in the tumor or for which the tumor variant allele fraction was <5 times than that of the normal variant allele fraction were disregarded [37]. 157 putative somatic mutations passed the filters (Table 1).

FACETS [38] was used to define copy number alterations. Specifically, read counts for positions within the target regions with dbSNP (Build 142) entries were generated for each matched tumor nodule and normal samples as input to FACETS, which performs a joint segmentation of the total and allelic copy ratio and infers allele-specific copy number states. To enable detection of the intragenic deletions of Tsc1 and Pten, 15-20 evenly-spaced positions per deleted exon were tiled within the regions of the deletions (Fig. S1).

### Transcriptome sequencing and quantitative PCR (qPCR) analysis

Raw fastq files were aligned to the reference genome Mus_musculus.GRCm38.72 using PALMAPPER with default parameters [39]. The length of the seeds of the PALMAPPER index was set to 15. Then we computed read counts using htseq-count against the reference genome annotation (Mus_musculus.GRCm38.72.gtf). Based on these counts we performed the differential gene expression analysis using DESeq2 where we compared each tumor sample individually against all six control samples. Exact numbers of detected dysregulated mRNA per tumor sample are given in Table 6. For quantitative PCR analysis, RNA was prepared as shown above, 500ng RNA was used to make cDNA using Superscript III (Invitrogen) as per manufacturers instructions. ABI Step One (Applied Biosystems) machine was used together with Syber Green PCR Kit (Invitrogen) and the primers below (100pM) to perform qPCR as per manufacturer instructions. TBP was used as a normalizer and ddCT method was used for analysis. Primer sequences:

**Table 6:** Number of upregulated, downregulated and unchanged mRNA, miRNAs, proteins and phosphosites per tumor nodule

	RNA			PROTEOME			PHOSPHOPROTEOME			miRNA		
MOUSE|NODULE	DOWN	NOT	UP	DOWN	NOT	UP	DOWN	NOT	UP	DOWN	NOT	UP
**Mouse1-N1**	1621	18953	2158	867	2029	1651	413	850	899	37	1243	51
**Mouse1-N2**	2269	18208	2651	979	2111	1328	407	1803	1701	60	1212	59
**Mouse1-N3**	1785	18786	2394	681	2117	1155	314	808	619	55	1234	42
**Mouse2-N1**	1909	18674	2473	1249	2568	1407	283	335	428	46	1234	51
**Mouse2-N2**	1692	19053	2310	774	1786	1653	887	1125	815	54	1235	42
**Mouse2-N3**	1950	18843	2559	860	2269	1310	251	1131	1299	49	1225	57
**Mouse3-N1**	2005	18645	2404	627	2959	715	294	1021	1255	49	1240	42
**Mouse3-N2**	2211	18392	2609	1228	2151	1933	286	411	514	59	1218	54
**Mouse3-N3**	1862	18803	2326	986	2028	1849	158	505	1019	51	1228	52
**Mouse4-N1**	1919	18640	2532	950	2128	1256	323	1759	1239	53	1210	68
**Mouse4-N2**	1538	19196	2275	840	2019	1588	300	1811	1461	62	1197	72
**Mouse4-N3**	2034	18319	2689	1070	2096	1715	454	1345	1185	70	1196	65

For each nodule of each mouse the number of upregulated, downregulated and unchanged mRNAs, miRNAs, proteins and phosphosites are given.

TBP F: ATGATGCCTTACGGCACAGG; R: GTTGCTGAGATGTTGATTGCTG;

CYR61 F: TAAGGTCTGCGCTAAACAACTC; R: CAGATCCCTTTCAGAGCGGT;

KI67 F: CGCAGGAAGACTCGCAGTTT; R: CTGAATCTGCTAATGTCGCCAA

CTGF F: GGCCTCTTCTGCGATTTCG; R: GCAGCTTGACCCTTCTCGG

BIRC5 F: GAGGCTGGCTTCATCCACTG; R: ATGCTCCTCTATCGGGTTGTC

CYCE1 F: CTCCGACCTTTCAGTCCGC; R: CACAGTCTTGTCAATCTTGGCA

### miRNA sequencing

miRNA sequencing libraries were generated using a modified protocol from [40]. Briefly, RNA from tissues was isolated using the Qiagen miRNAeasy kit as described above (section Animal experiments). 10 microgram of total RNA was run in a 15% polyacrylamide gel, the part containing small RNAs was cut and subjected to nucleotide extraction using overnight 0.4M NaCl and ethanol precipitation. Isolated small RNA mix was subjected to Illumina TrueSeq Small library preparation kit used as per manufacturer’s instructions. Afterwards the small-RNA libraries were run in a 10% Polyacrylamide gel to clean up the adaptor-adaptor fraction. The gel part containing the small-RNA libraries was cut and libraries were extracted using overnight 0.4M NaCl and ethanol precipitation. They were run using Illumina NextSeq500 sequencer as per manufacturer’s instructions. Exact numbers of detected dysregulated miRNA per tumor sample are given in Table 6.

### Mass spectrometry for proteomics and phosphoproteomics

Liver tissues from L-dKO tumors and control mice were obtained as detailed above (Animal experiments). Label free mass spectrometry was performed on the tumor nodules. Tumor proteome was always compared to the proteome obtained from livers of six control mice pooled together. A detailed description about the proteomics method used to analyse L-dKO tumor nodules and the softwares used for data analysis can be found in [2]. For the phosphoproteome, the desalted peptides were enriched for phosphopeptides using TiO2 beads. Detailed protocol is available in [3]. After data processing, the protein groups datasets and phospho peptide datasets were exported into a FileMaker Pro-12 databank. For statistical analysis, an R-based program - Perseus, version 1.4.0.2, was used [41]. ANOVA-based two-sample t-test was performed by adjusting S0 to 1 and the number of randomizations to 250 (default). The 5% FDR was used for analysis. Exact numbers of detected dysregulated proteins and phosphosites per tumor sample are given in Table 6.

### Detection of differentially expressed mRNA and miRNA

The DESeq2 tool [42] with default settings was used to detect differentially expressed genes and miRNA between tumor and normal tissue. Every tumor sample was compared against the six control samples from healthy liver tissue. We considered as significant the genes detected with an FDR adjusted *P*-value lower than 0.05.

### Interaction network

In order to construct a directed functional network, we downloaded functional interactions for the species Mus Musculus from three different databases including Kegg, Signor and miRTarBase. From miRTarBase, we only kept the interactions supported by strong experimental evidence (either reporter assay or western blot). The interactions cover a variety of types at different cellular levels, including (de) phosphorylation (phosphoproteome), expression/repression (RNA) and activation/inhibition (proteome). Interactions characterized as “binding” or “complex” were treated as undirected edges. The network contained 5,546 genes and 44,423 interactions in total. In order for network diffusion to converge to a unique solution (steady state), we only used the largest strongly connected component of the network, which contains 2,484 genes and miRNAs and 32,954 interactions. We excluded self-interactions.

### NetICS

We employed NetICS [11] for data integration and network gene ranking. We used differentially expressed miRNAs and somatic mutations (SNV, indels) as upstream causal events. miRNA differential expression was computed in a sample-specific manner by comparing each tumor nodule to the 6 control samples from healthy liver tissue. As downstream events, we used differentially expressed genes/proteins at the RNA, proteome and phosphoproteome levels. At the phosphoproteome level, one gene was included if there was at least one differentially expressed phosphosite in its protein when tested between tumor and normal tissue. Data at the downstream level were integrated by using the rule described at Table 7.

**Table 7:** Combination rules for differentially expressed genes

Combinations		
RNA	PROT or PH	Output
Significant/Insignificant	Significant	PROT or PH
Significant/Insignificant	Insignificant	Not taken
Significant	Not Detected	RNA
Insignificant	Not Detected	Not taken
Not Detected	Not Detected	Not taken

The genes at the downstream level are combined as follows: If the protein is significantly changed at the proteome or phosphoproteome levels, it is taken into account in the set of differentially expressed genes/proteins given as input in NetICS. If the protein is detected but not significantly changed at the proteome or phosphoproteome levels, it is not taken into account. If the protein or its phosphosites are not detected at all, then the change at the RNA level is taken into account.

After we run NetICS, we kept the top 5% of the genes in the ranked list. We performed a random permutation test by permuting the labels of differentially expressed genes, miRNAs and mutated genes for each sample. We then recomputed the gene list and computed an empirical *p*-value for each gene by counting how many times the score given by NetICS was higher than the original score. We repeated the random permutation procedure 10,000 times and adjusted the *p*-value by FDR correction [43]. We ended up with 74 genes in total.

### Antibodies

Yap1 ((G-6) sc-376830), HDAC4 (CST 7628), Sirt1 (CST 3931) and total AKT (CST, 9272) were obtained commercially. Horseradish peroxidase (HRP)-coupled anti-mouse (115-035- 774) and anti-rabbit (211-032-171) secondary antibodies were purchased from Jackson laboratories.

### Immunoblotting

Both human and murine liver tissue was homogenized in T-PER (ThermoFisher scientific, 78510) supplemented with 1 mM PMSF, 1× Complete Mini Protease Inhibitors (Roche), 1× PhosSTOP (Roche) using a Polytron (PT 10-35 GT) at 500*g* for 2 min. Equal amounts of homogenate were SDS–PAGE fractionated and transferred onto a nitrocellulose membrane that was incubated, after blocking (5% BSA in TBST), with appropriate antibodies.

### Source of human samples

All human samples used in this study were obtained after following the relevant ethical regulations. An informed consent was obtained from the human subjects.

## Supplementary Information

**Additional file 1: Figure S1.** Copy number profiles derived from whole-exome sequencing demonstrates the intragenic deletions of *Tsc1* and *Pten*. For each nodule, segmented Log2 ratios (y-axis) were plotted according to their genomic positions (x-axis), for chromosomes 2 or 19. Red arrows indicate the loci of the intragenic deletions of *Tsc1* and *Pten*.

**Additional file 2: Figure S2. A.** Graph showing miRNA expression of miR-375 in L-dKO tumors (*n*=12) compared to control mice (*n*=6). **B.** mRNA expression analysis of indicated genes in 20-week-old L-dKO tumors compared to livers from age-matched control mice (*n* = 6). Expression for each gene is normalized to intensity of *TBP* gene expression (normalising control) in the corresponding mice. Two-sided unpaired *t*-test is used. Data is mean ± s.d.

**Additional file 3: Figure S3.** Graph showing the expression of miRNAs (upstream of HDAC4) in L-dKO tumors (*n*=12) compared to control mice (*n*=6).

**Additional file 4: Figure S4.** Graph showing the expression of miRNAs (upstream of SIRT1) in L-dKO tumors (*n*=12) compared to control mice (*n*=6).

**Additional file 5: Figure S5.** Original western blots full length images of the images shown in Figures 5A and C.

## Data Availability

The DNA and RNA raw murine data analyzed in this study is available in the Sequence Read Archive repository, under the accession SRP156216 [https://www.ncbi.nlm.nih.gov/sra/?term=SRP156216]. The raw proteomic and phosphoproteomic murine data as well as the interaction network used for the analysis are available in our github repository [https://github.com/cbg-ethz/netics/tree/master/mouse_data]. The reference mouse genome GRCm38 was downloaded from [https://www.ncbi.nlm.nih.gov/assembly/GCF_000001635.20/].

## Abbreviations

HCC: Hepatocellular carcinoma
NetICS: Network-based integration of multi-omics data
miRNA: Micro RNA
mRNA: Messenger RNA
L-dKO: Liver-specific double-knockout
PCR: Polymerase chain reaction
qPCR: Quantitative polymerase chain reaction
FDR: False discovery rate
SNV: Single nucleotide variant
Indel: Insertion/deletion
EMT: Epithelial to mesenchymal transition
TCGA: The cancer genome atlas
