# Supplementary figures and images for: Multi-omics data integration reveals novel drug targets in hepatocellular carcinoma
Source: BMC Genomics. 2021 Aug 4;22:592. doi: 10.1186/s12864-021-07876-9 (PMC8340535; doi:10.1186/s12864-021-07876-9)

Figure S4

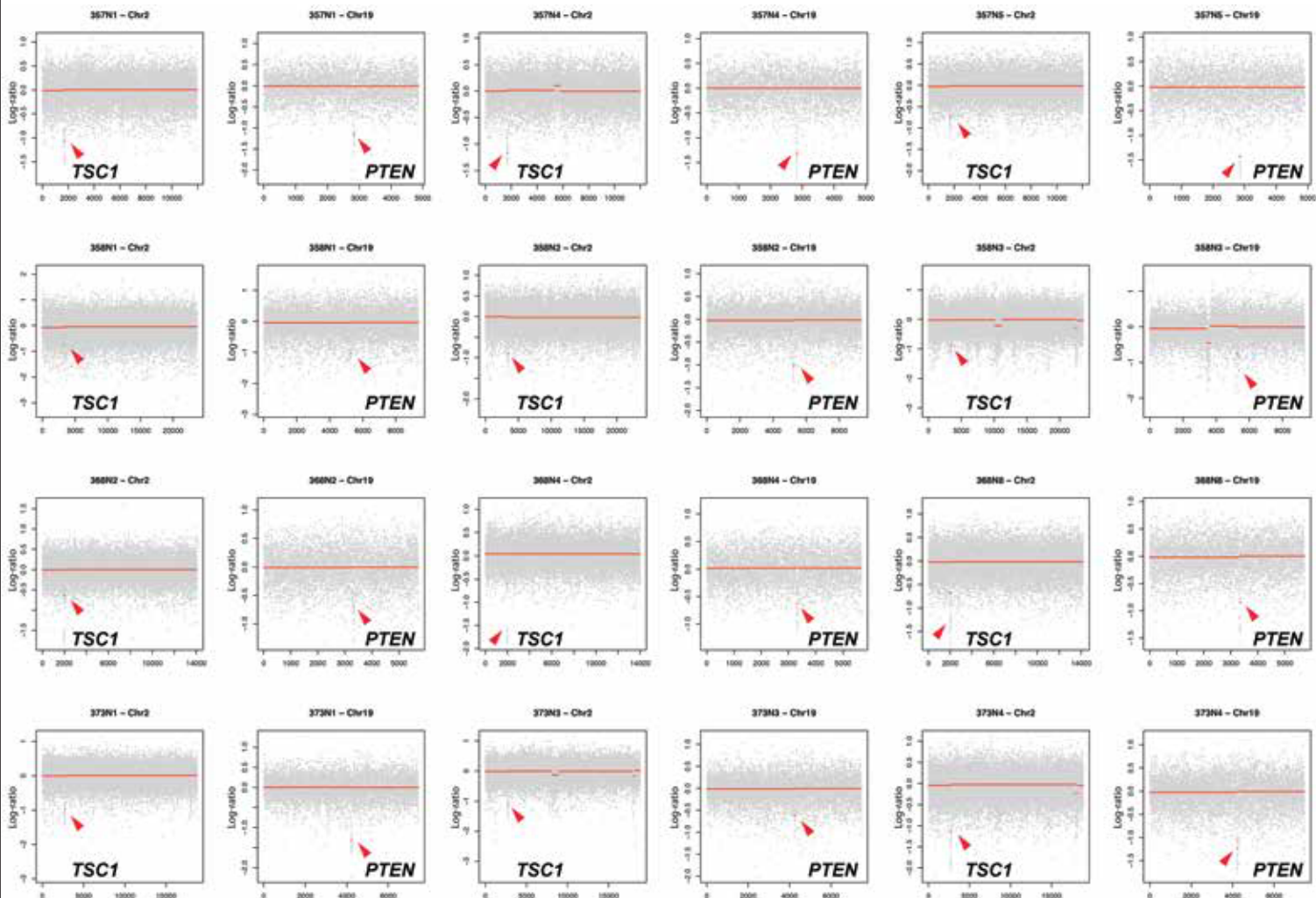

Supplement: Supplementary file 1 — Additional file 1: Figure S1. Copy number profiles derived from whole-exome sequencing demonstrates the intragenic deletions of Tsc1 and Pten. For each nodule, segmented Log2 ratios (y-axis) were plotted according to their genomic positions (x-axis), for chromosomes 2 or 19. Red arrows indicate the loci of the intragenic deletions of Tsc1 and Pten. [file 12864_2021_7876_MOESM1_ESM.pdf]

**Figure S2**

**A.**

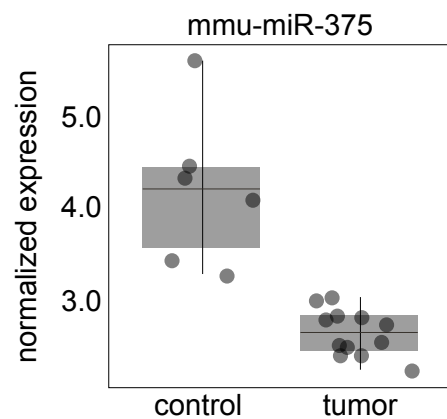

**B.**

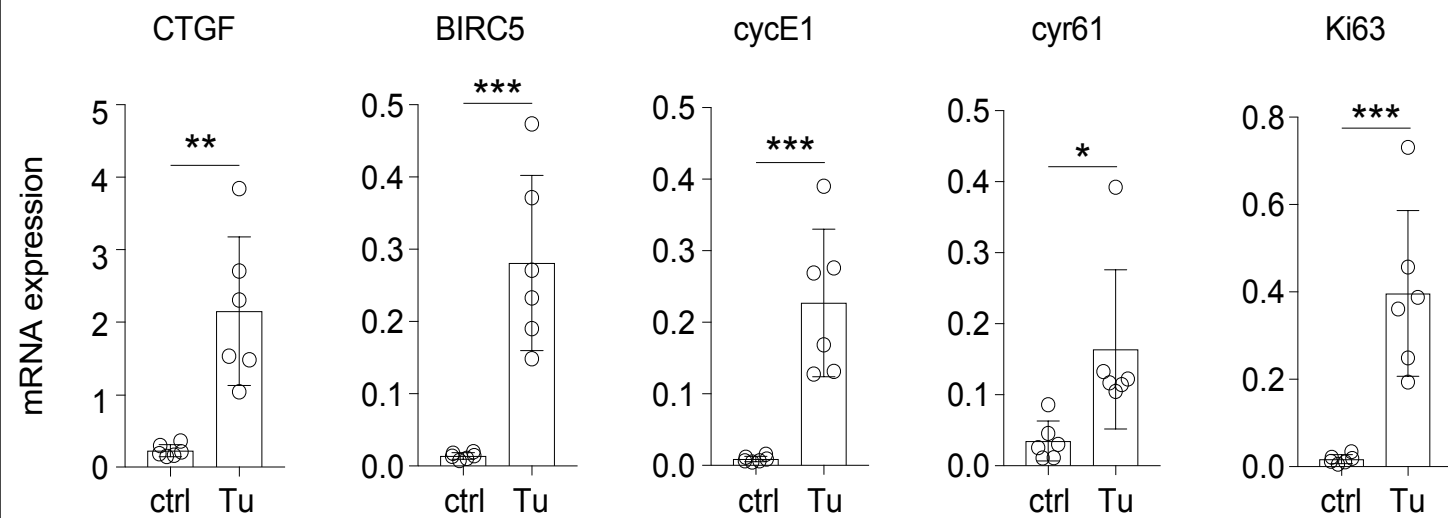

Supplement: Supplementary file 2 — Additional file 2: Figure S2. A. Graph showing miRNA expression of miR-375 in L-dKO tumors (n=12) compared to control mice (n=6). B. mRNA expression analysis of indicated genes in 20-week-old L-dKO tumors compared to livers from age-matched control mice (n = 6). Expression for each gene is normalized to intensity of TBP gene expression (normalising control) in the corresponding mice. Two-sided unpaired t-test is used. Data is mean ± s.d. [file 12864_2021_7876_MOESM2_ESM.pdf]

**Figure S3**

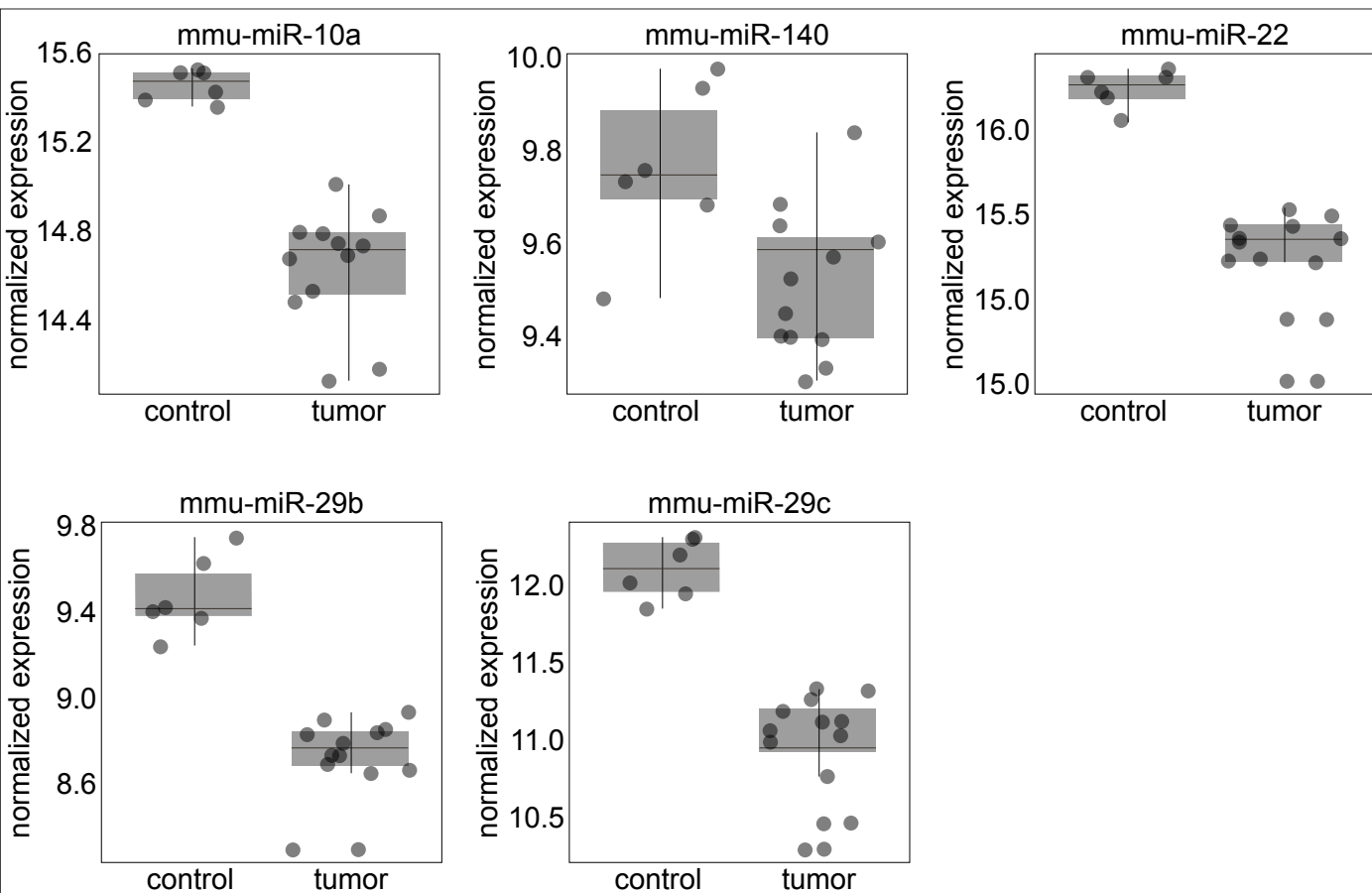

Supplement: Supplementary file 3 — Additional file 3: Figure S3. Graph showing the expression of miRNAs (upstream of HDAC4) in L-dKO tumors (n=12) compared to control mice (n=6). [file 12864_2021_7876_MOESM3_ESM.pdf]

**Figure S4**

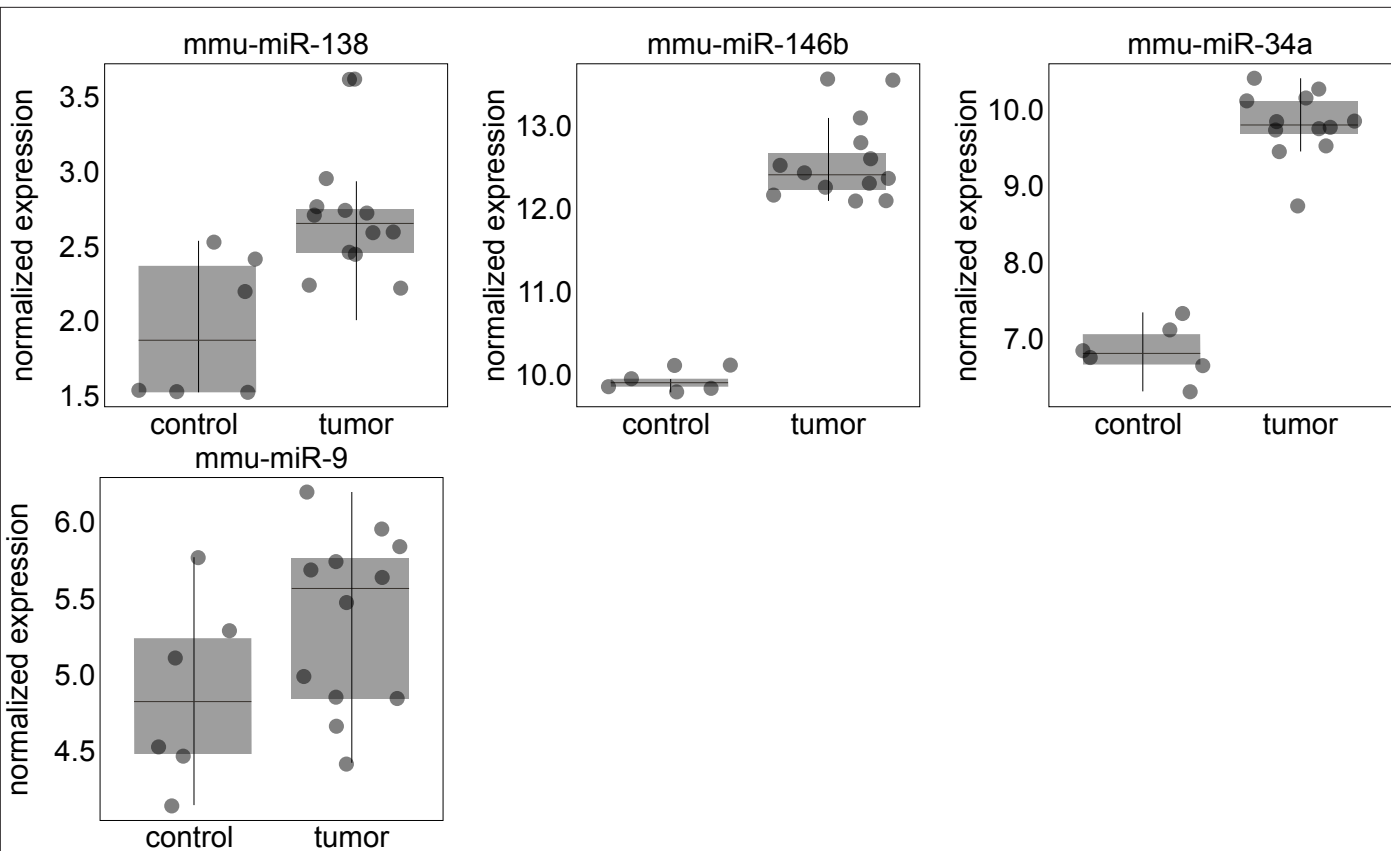

Supplement: Supplementary file 4 — Additional file 4: Figure S4. Graph showing the expression of miRNAs (upstream of SIRT1) in L-dKO tumors (n=12) compared to control mice (n=6). [file 12864_2021_7876_MOESM4_ESM.pdf]

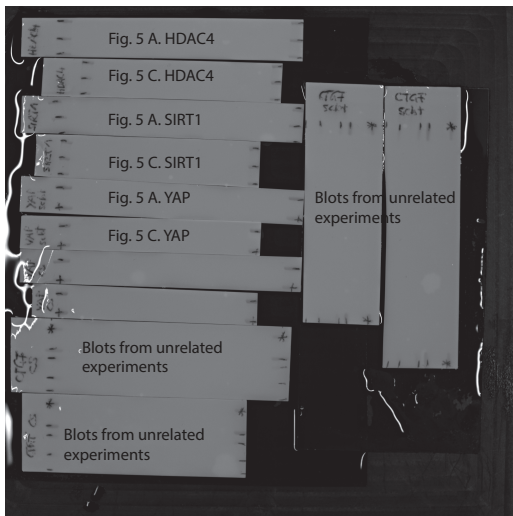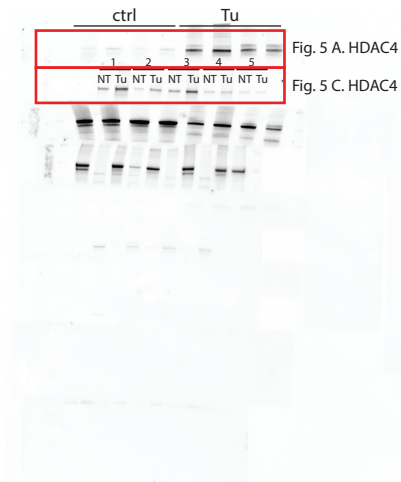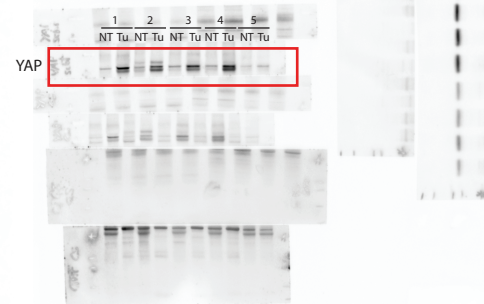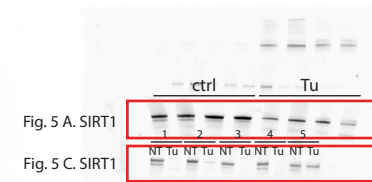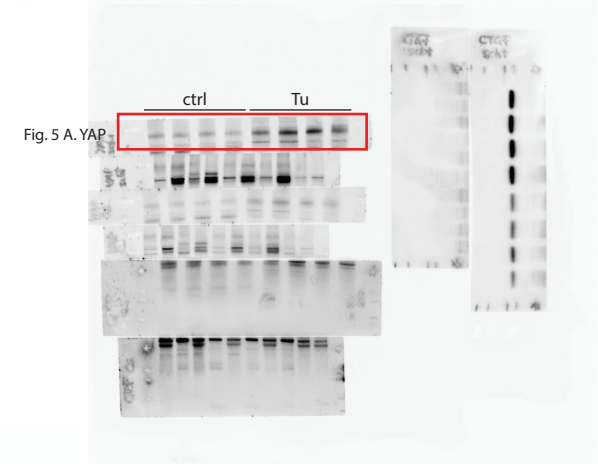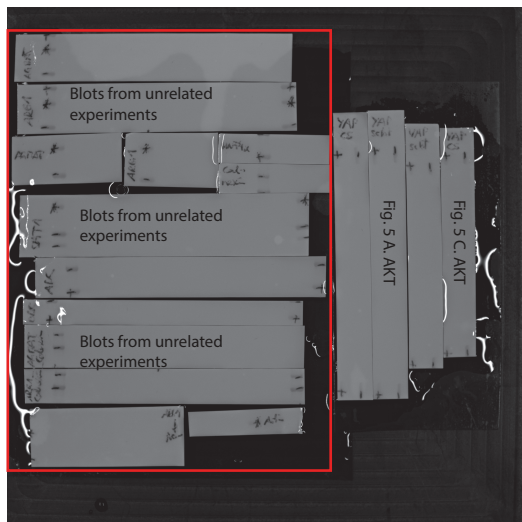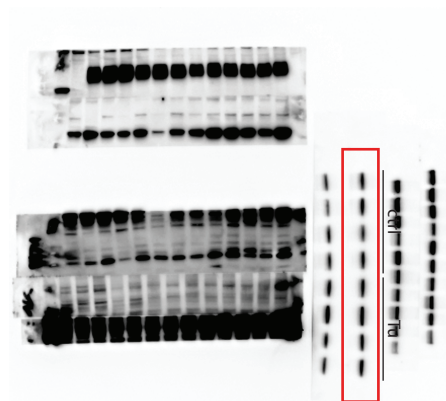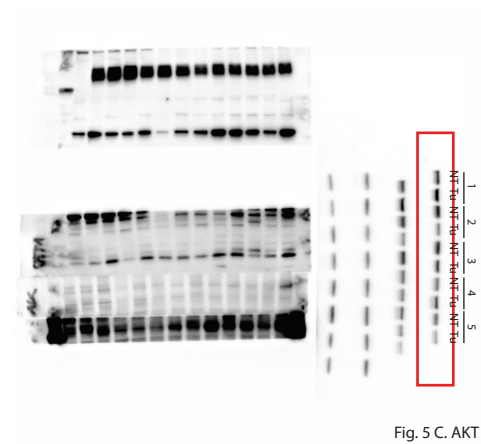

YAP membranes stripped and re-probed for AKT

Supplement: Supplementary file 5 — Additional file 5: Figure S5. Original western blots full length images of the images shown in Figures 5A and C. [file 12864_2021_7876_MOESM5_ESM.pdf]
